# Quality of care for non-communicable diseases in the Republic of Moldova: a survey across primary health care facilities and pharmacies

**DOI:** 10.1186/s12913-019-4180-4

**Published:** 2019-06-04

**Authors:** Carolyn Blake, Leah F. Bohle, Cristina Rotaru, Natalia Zarbailov, Valeriu Sava, Florence Sécula, Helen Prytherch, Ala Curteanu

**Affiliations:** 1 Swiss Centre for International Health, Swiss Tropical and Public Health Institute, Basel, Switzerland; 2 University of Basel, Basel, Switzerland; 3 Healthy Life project: Reducing the Burden of Non-Communicable Diseases in Moldova, Chisinau, Moldova; 4 Swiss Agency for Development and Cooperation (SDC), Chisinau, Moldova; 5 State University of Medicine and Pharmacy “Nicolae Testemiţanu”, Chișinău, Moldova; 6 Mother and Child Institute, Chisinau, Moldova

**Keywords:** Quality of care, Health systems, Primary health care, Service coverage, Non-communicable disease, Accountability, Moldova, Health facility survey, Family medicine, Universal health coverage

## Abstract

**Background:**

The Republic of Moldova is faced with a high prevalence of non-communicable diseases (NCDs) related to lifestyle and health behavioural factors. Within the frame of the decentralisation reform, the primary health care system has been tasked to play an important role in the provision of preventative and curative NCD health services. There is however limited evidence available on the actual coverage and quality of care provided. Our paper aims to provide an updated overview of the coverage and quality of service provision in rural and urban regions of Moldova.

**Methods:**

We designed a facility-based survey to measure aspects of coverage and quality of care of NCD services across 20 districts of the Republic of Moldova. This study presents descriptive data on the structural, procedural and clinical aspects of primary healthcare delivery at health centre and family doctor office level. Adjacent private pharmacies were also assessed for the availability of essential NCD medicine.

**Results:**

Organised under the WHO Health Systems Framework, our findings highlight that service provision and information were generally the strongest among the six health systems building blocks, with more weaknesses found in the area of the health workforce, medical products, financing, and leadership/governance. Urban facilities generally fared better across all indicators.

**Conclusions:**

The gaps in service provision identified by this study require broad health system improvements to ensure NCD related policies and strategies are embedded in primary health care service provision. This likely calls for stronger coordination and collaboration between the public and private sectors and the different levels of government working towards ensuring universal health coverage in Moldova.

**Electronic supplementary material:**

The online version of this article (10.1186/s12913-019-4180-4) contains supplementary material, which is available to authorized users.

## Background

The Republic of Moldova is faced with a high prevalence of non-communicable diseases (NCDs) related to lifestyle and health behavioural factors [1]. The 2013 STEPS survey undertaken by the World Health Organisation (WHO) highlighted that every third person in Moldova (30.3%) had three or more risk factors for NCDs, with this figure increasing proportionally with age. Bearing the highest general mortality rate in the European Region, NCDs account for 85.6% of deaths annually in the country [1]. NCDs with the highest mortality rates are cardiovascular diseases (CVDs), chronic respiratory diseases, diabetes and cancer [1].

In the last decade, the Government of the Republic of Moldova has taken important steps to tackle NCDs, and their risk factors, through the adoption of numerous national strategies, policies for the prevention and control of NCDs (i.e. the National Strategy for Prevention and Control of Non-Communicable diseases 2012–2020) as well as a number of intersectoral or disease specific programmes, [2]. These efforts are embedded in broader health systems strengthening reforms underway in the last two decades that aim to reduce health inequalities and achieve Universal Health Coverage (UHC) [3]. A major part of the reform has been the institutionalisation of a decentralised primary healthcare (PHC) system based on a Family Medicine model. This has gradually shifted financial resources from tertiary to primary health care – mainly to health centres (HCs) and family doctor’s offices (FDOs) [2]. Under this reform, policies emphasise the importance of prioritising health promotion and disease prevention in PHC, strengthening health service delivery in rural areas, and strengthening the quality of services [4]. The introduction of a Mandatory Health Insurance (MHI) in 2004 has also been an important reform component Under the MHI government approved service package, basic primary health care and emergency medical services are provided free of charge regardless of insurance status [5]. However, only insured individuals (approx. 74%) are covered for inpatient hospital services and specialised outpatient services [6, 7]. In addition, only a limited number of medicines - including for NCDs - are fully or partially compensated under the MHI or via disease-specific programmes [7]. The cost of medicines is the main reason for high Out-Of-Pocket Payments (OOPs) in Moldova (approx. 73.5%), with some essential medicines not included under the list of reimbursable drugs [3].

To date, available data and studies on the current state of primary health care delivery and more specifically for NCDs, have mainly focused on health systems and policy related issues [4, 8, 9] with a focus on the MHI [5, 10, 11], and OOPs [6, 12] and their effect on access to healthcare. Some studies have also focused on the acceptability of healthcare, by collecting patient perceptions on quality of care [1, 4, 13].

There is however limited evidence available on the actual coverage and quality of care delivered in healthcare facilities. The last primary health-care assessment was carried out in 2007 and focused mainly on infrastructure-related aspects (facility, equipment and inputs) [14]. The study reported shortages of 40–90% of basic health facility equipment (according to national norms). The majority of the lowest ranking health centres were located in rural areas of the country. In 2012, the WHO noted that a follow-up study was needed to assess progress made since 2007, especially on the availability and management of screening and prevention services for non-communicable diseases at primary healthcare level, and the quality of care delivered including compliance with clinical protocols [15]. In terms of medical products, a 2011 WHO study assessed urban and rural pharmacies in terms of the price, availability and affordability of essential medicines. Findings highlighted significant gaps in medicine availability, especially in rural area pharmacies [16].

Our paper aims to provide an updated overview of the NCD related services at primary health care level in Moldova. This study was carried out in the framework of the Healthy Life Project in Moldova: ‘Reducing the Burden of NCDs 2016–2020’, funded by the Swiss Agency for Development and Cooperation (SDC). We aimed to assess the current state of the availability and quality of NCD services in Moldova at health centre, family doctor office and pharmacy level. The study focuses on NCD service provision in general; however, to collect more in-depth information, data collection partly focused on three tracer diseases: hypertension, ischemic heart disease, diabetes. The study’s tracer diseases were selected based on the project’s focus, and their high prevalence in the country (diabetes is considered to be underreported in the country). Diabetes prevalence: 8.4% of women and 10.1% of men [3]; prevalence of hypertension is 40.3% for men and 39.3% for women [1]. Cardiovascular diseases account for first cause of mortality in Moldova (59% of all deaths) with ischemic heart disease at the top of the ranks [3].

### Framework

**Fig. 1:**
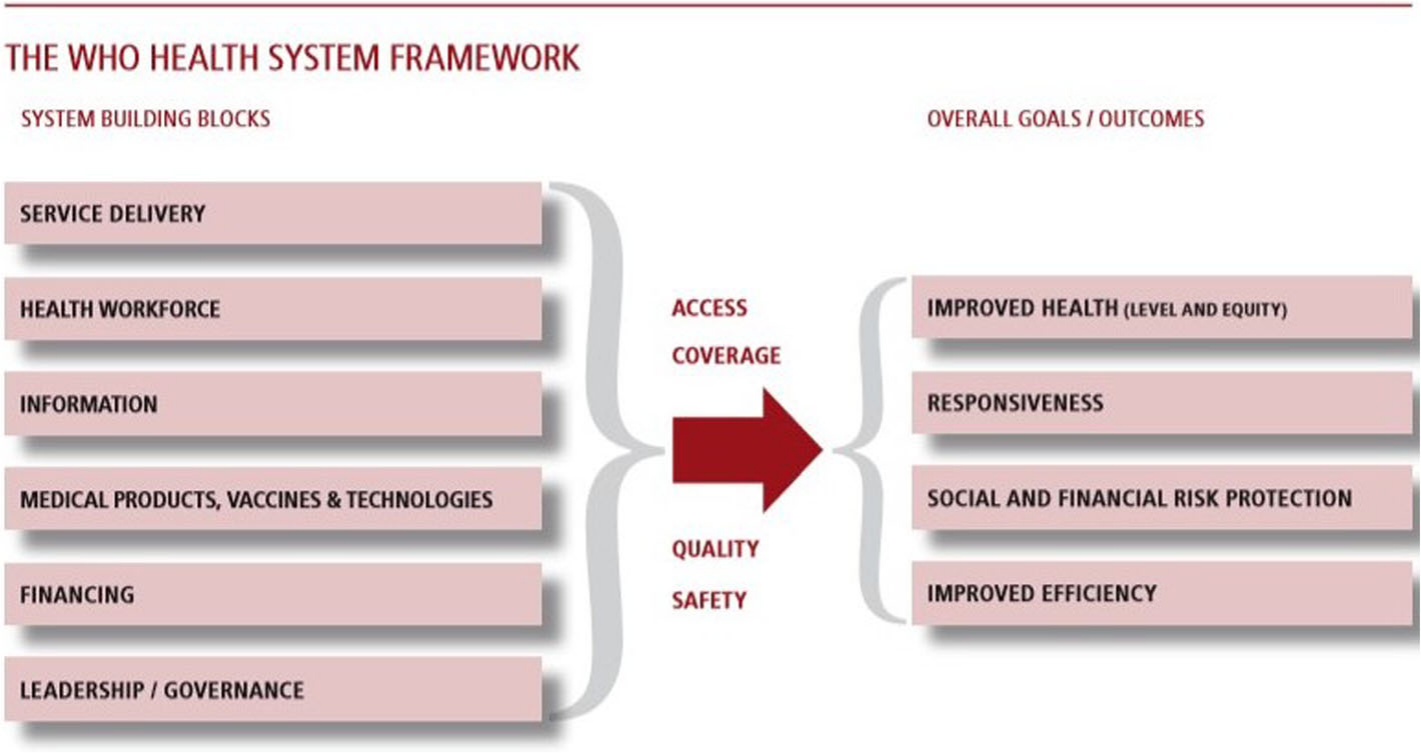
WHO Health Systems Framework

The results of this study are organised and presented around the WHO Health Systems Framework, and its systems building blocks [17]. According to this framework, the six building blocks of a health system (namely service delivery; health workforce; information; medical products, vaccines and technology; financing; leadership/governance) all influence health service access, coverage, quality and safety, which consequently have an effect on health (level and equity), responsiveness, social and financial protection as well as improved efficiency. The building blocks and outcomes will be discussed throughout the paper in light of our findings and speak to SDG target 3.8 “Achieve universal health coverage, including financial risk protection, access to quality essential health-care services and access to safe, effective, quality and affordable essential medicines and vaccines for all”. See Fig. 1. This facility survey however can only provide evidence on the status of the building blocks, and do not intend to measure health systems related outcomes.

## Methods

### Aim, design and setting of the study

We designed a facility-based survey to measure aspects of coverage and quality of care of NCD services across 20 districts of the Republic of Moldova. Structural, procedural and clinical aspects of primary healthcare delivery were assessed at the level of primary healthcare centres (PHC) and Family Doctor Offices (FDOs). Local pharmacies were also assessed for availability of NCD medicines (at least partially compensated by medical insurance) for the three tracer diseases. Although this study is part of a broader controlled survey, the present paper only presents and discusses results of the baseline survey. Thus, this paper only provides descriptive data and does not intend to evaluate any policies or programmes. Data is presented through comparison groups: health centres and family doctor offices, and the North, South, Centre regions of the country.

### Characteristics of participants or description of materials

The study included three modules: (a) infrastructure, services and processes, (b) consultation observations, and (c) a retrospective medical record review of hypertensive patients. The modules are based on key documents and surveys from the World Health Organisation (WHO) [1, 8, 18], other project surveys from the region [18–21], and tailored to the Moldovan context including national PHC/NCD related norms and policies and national clinical protocols [22, 23]. The tools were translated into Romanian and Russian and back-translated into English to ensure translation quality.

In total there were 472 study participants, including PHC/FDO facility in-charges (doctors), family doctors, pharmacists as well as patients attending a consultation for one of the tracer diseases. In total, 60 health facility staff (survey module a), 54 pharmacists (survey module b) were interviewed (survey module a); and 182 patients (ages 18–69) were observed during consultations (survey module c). All survey questionnaires are available as additional files (See Additional file 1: survey module a; Additional file 2: survey module b; Sdditional file 3: survey module c). Survey module (a) comprised of interviews with facility in charges, and direct observation for infrastructure, services and processes by the data collector; survey module (b) entailed interviews of pharmacists and direct observation of availability of specific medicine; survey module (c) involved the direct observation of the clinical consultation. This study complies with STROBE guidelines on reporting of observational studies.

### Sampling process

**Fig. 2:**
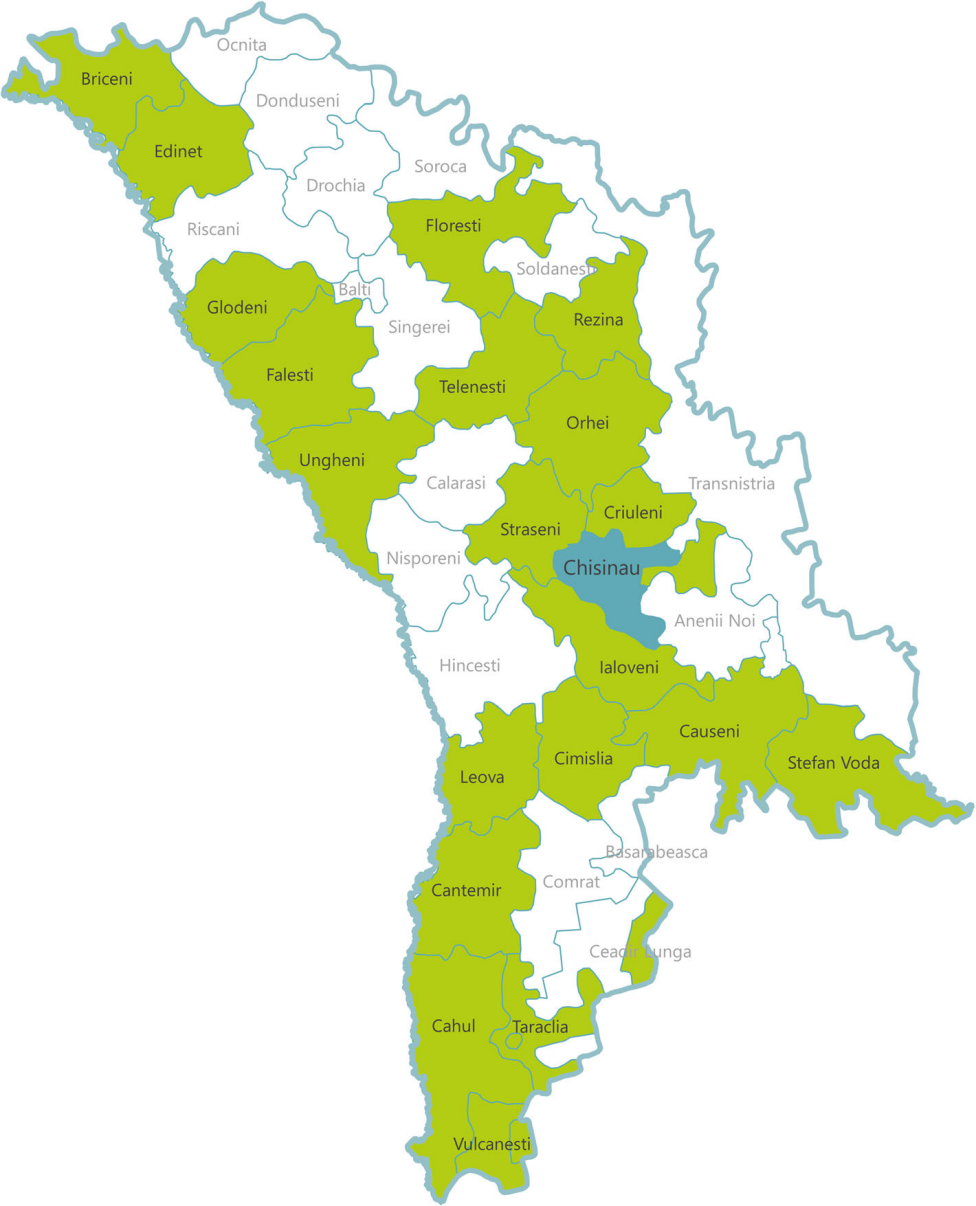
Map of study districts

Based on the list of the ten intervention districts of the Healthy Life project, another 10 neighbouring districts were selected as controls based on proximity and comparative size. However as this is only the baseline survey, results are not presented by intervention/control regions. In total, 60 facilities and 60 pharmacies were selected at random from the 20 districts (See Fig. 2. Map of study districts). The sample includes urban and rural PHCs/FDOs that had more than 2′000 residents registered with the centre/office - according to the National Center of Health Management data. The selection criteria were as follows: depending on the total population of the district, one to four HCs/FDOs were sampled per district to ensure sufficient data for the needs of the three survey modules. In each district at least one urban HC, one rural HC and one rural FDO were included, except for districts with a small population where less than three collection points were required. Furthermore, selected FDOs were not in the catchment area of the selected HC, and when possible, at least one permanent doctor and one facility manager was on staff, according to the latest available MoH annual report.

In Moldova, a rural area is defined as a setting where the majority of the labour force is working in agriculture, forestry and fishing. An urban area is where the majority of the labour force is working in non-agriculture branches of economy, including industries and services (law nr.764, 27.12.2001).

The study findings are divided into 3 main geographical regions: North, Centre and South. These regions are not administrative units, but are considered as functional territorial units used for the planning, evaluation and implementation framework of the country’s regional development policy.

Probability proportional to size (PPS) sampling method was applied for the selection of sampling units, as recommended by the WHO STEP-wise approach to non-communicable disease risk factor surveillance methodology (STEPS) [24]. This allowed each eligible facility a proportionate chance of being sampled. Using the STEPS sampling tool [25], we selected at random the defined number of facilities per district.

In each HC/FDO, three consultations were observed of patients with one of the following three tracer conditions. In total, 180 observations were planned. The consultations were observed from up to three different doctors. We aimed to observe one consultation per tracer disease per facility, but when this was not possible, 3 consultations of any of the three tracer diseases were carried out.

Our final sample included 60 public facilities (16 HCs, 44 FDOs) with a 100% response rate, 54 adjacent private pharmacies (closest to HC/FDO) (90% response rate), 182 observations of consultations for hypertension, ischemic heart disease, diabetes (99.4% response rate). In total, there were 20 urban HCs, 24 rural HCs and 16 rural FDOs. Response rates were high as the survey coordinator was able to work with districts to book appointments with health facilities in advance. Clinical observations were also high as data collectors would spend one day in localities thus ensuring enough time to attend consultations.

### Ethics

Ethical clearance was received from the National Ethics Committee of the Ministry of Health, Social Protection and Labour in Moldova. Reference number 339, 31.05.2017. Before data collection, relevant authorities and facility managers of selected facilities were informed about the study, its purpose and when data collection would take place. Participation was voluntary, and before taking part in the study, participants were asked for their verbal consent and given an information leaflet about the aims and objectives of study, as well as their rights as participants. Verbal consent was approved by the Ethics Committee due to this being a non-interventional study. No material or financial incentives were provided to participants. A list including the name of the respondents and the allocated identification numbers were kept separate to the data collected. The survey did not entail any invasive techniques or procedures.

### Pilot and data collection

A pilot test of the survey methodology, tools and translation was carried out in one health centre during the training period. However, this data was not included in the final data set. Based on pilot results, the tools were then refined, adapted and finalized based on the findings.

Data collection was carried out over a two-week period in June–July 2017, by four teams of two data collectors with a strong background in medical training and public health (*N* = 8). One member of each team was a supervisor. Data was collected via tablets using the Open Data Kit (ODK) software. On a daily basis, team supervisors as well as the national coordinator carried out quality assurance measures to ensure data completeness and fidelity to study methodology.

### Statistical analysis

The analysis process ensured full confidentiality of respondents. Data was analysed using the GNU PSPP software [26]. Whenever aggregating responses from multiple variables was necessary, cross-tables per variable were generated, the frequency for each combination of responses summed up and column percentages calculated. Comparison of groups was performed using Pearson’s chi-squared test (χ^2^) and analysis of variance (ANOVA).

## Results

The results of this study are organised and presented according to the WHO Health Systems Framework, and its systems building blocks. The results provide a limited overview of the state of the health systems building blocks in the Republic of Moldova, but do not cover indicators as per full WHO handbook [27] See Fig. 1.

### Service delivery

According to the WHO health system framework, good health services are those which deliver effective, safe, quality personal and non-personal health interventions to those who need them, when and where needed, with minimum waste of resources" [17].

#### Availability of essential NCD-related services, clinical protocols and health promotion materials

The availability of preventative and curative PHC services was assessed based on the national list of services from the Ministry of Health. For the purpose of this study, we are only reporting on NCD related services, which account for 4 /9 preventative PHC services and 7/11 curative PHC services. See Table 1 below.

**Table 1:** Essential primary health care services related to NCDs

Essential preventative PHC services -related to NCDs	
• Promoting healthy lifestyles, inculcating skills in preventing and combating risk factors	
• An annual medical examination with a prophylactic examination of persons over the age of 18 with the aim of preventing	
• Periodic medical examinations of patients with diseases registered with a family doctor;	
• Provision of services for early detection of pathology based on screenings	
Essential curative PHC services -related to NCDs	
• NCD consultation (primary examination, clinical examination, diagnosis, treatment)	
• Medical treatment (intramuscular, intravenous procedures)	
• Medical and hygienic-dietary treatment, including compensated medicines and medical products	
• Monitoring of patients according to treatment plan	
• Referral to other services such as laboratory rapid tests, as well as physiotherapy and physical rehabilitation services	
• Referral to a specialized physician in cases that are beyond the competence of the family doctor	
• Medical care at home	

Results show that facilities reported a high availability of essential services related to NCDs, with only a few facilities (*N* = 4/60) mentioning service gaps in‘ early detection of pathology based on screenings’, as well as in ‘medical care at home’. Updated clinical protocols for PHC and the three tracer diseases were available in all facilities (*N* = 60)–either in their paper or electronic versions: A majority of health facilities had access to both versions (HC: 70.45%; FDO: 62.50%). Health promotion materials (posters, brochures, leaflets) were present in 88.64% of HCs and only 56.25% of FDOs, a statistically significant difference (χ^2^ = 7.692, *p* = 0.006). Leaflets on smoking, alcohol consumption, nutrition, hypertension and diabetes were the most commonly available. In about a third of FDOs, no health promotion materials were present at all. See Table 2 below.

**Table 2:** Service Delivery (±SD, *p < 0.05,**p < 0.01, ***p < 0.001)

Service delivery	Facility type		Region			N
	HC	FMO	North	Centre	South	N = 60
Score of availability of preventative NCD services (mean, out of 4)	3.84 ± 0.53	3.88 ± 0.34	3.72 ± 0.75	3.95 ± 0.22	3.86 ± 0.36	60
Score of availability of curative NCD services (mean, out of 7)	6.93 ± 0.25	6.81 ± 0.54	6.83 ± 0.51	6.90 ± 0.30	6.95 ± 0.22	60
Availability essential national clinical protocols are available (%)						
Clinical protocols on Hypertension	100.00%	100.00%	100.00%	100.00%	100.00%	60
Clinical protocols on Ischemic Heart Disease	97.73%	93.75%	100.00%	95.24%	95.24%	58
Clinical protocols on Diabetes	100.00%	93.75%	100.00%	95.24%	100.00%	59
Availability of health promotion materials for patients (leaflets, brochures) (%)	93.18%	68.75%	72.22%	85.71%	100%	52
Visibility and accessibility of facility (%)						
The facility location is visibly displayed in locality	88.64%**	56.25%**	77.78%	85.71%	76.19%	48
Opening hours are visibly displayed to the public	97.73%**	75.00%**	88.89%	90.48%	95.24%	55
Staff contact phone numbers are visibly displayed to the public	93.18%**	62.50%**	77.78%	85.71%	90.48%	51
Tariffs visibly displayed to the public/patients	54.55%*	25.00%*	50.00%	33.33%	57.14%	28
Availability of infrastructure, cleanliness and maintenance (cumulative score, out of 17) (%)	89.96%	82.46%	85.20%	89.08%	89.27%	60
Availability of water, sanitation and hygiene (cumulative score, out of 17) (%)	89.04%	81.34%	85.20%	85.35%	90.20%	60
Availability of referral mechanism for medical emergencies (%)	90.91%*	68.75%*	72.22%	85.71%	95.24%	51
Availability of laboratories in health facilities (%)	84.09%**	50.00%**	83.33%	71.43%	71.43%	45
Availability of at least one pharmacy in the locality offering compensated medicines (%)	95.45%**	68.75%**	94.44%	85.71%	85.71%	53
Availability of community outreach plans (available and used) (%)	63.64%	56.25%	44.44%*	52.38%	85.71%*	37

#### Visibility and accessibility of facility

Four indicators were used to assess the visibility and accessibility of facilities. Results highlight that health centres were more visible and accessible than FDOs: the facility location visibly displayed in locality (HC: 88.64%; FDO: 56.25%, χ2 = 7.692, p = 0.006); the visible display of staff contact phone numbers (HC: 93.18%; FDO: 62.50%, χ2 = 8.663, *p* = 0.003), of opening hours (HC: 97.73%; FDO: 75%, χ^2^ = 7.934, *p* = 0.005); and of tariffs to the public/patients (HC: 54.55%; FDO: 25%, χ^2^ = 4.115, *p* = 0.042). See Table 2.

#### Quality of infrastructure, overall cleanliness and maintenance

The overall infrastructure, cleanliness and maintenance was assessed based on a cumulative score calculated on a series of 17 variables (See Additional file 4: Infrastructure, cleanliness and maintenance). Results highlight that 89.96% of HCs and 82.46% FDOs scored positively on surveyed indicators. The main gaps revealed were the absence of printers and internet connection at FDO level, and the occurrence of power cuts (last 7 days) especially at health centre level (HC: 52.27%; FDO: 18.75%, χ^2^ = 5.370, *p* = 0.020).See Table 2 for overall scores.

In terms of water, sanitation and hygiene, a cumulative score was calculated based on a series of 17 indicators (See Additional file 5: Water, sanitation and hygiene). Results show that health centres scored slightly higher than FDOs (89.04, 81.34% respectively). Items most often missing were the availability of warm water, chlorine solution or other disinfectants to disinfect contaminated instruments, and the safe disposal of infectious waste. See Table 2 for overall scores.

#### Availability of referral mechanisms, laboratories and pharmacies

Referral of patients to other specialised physicians and other services was practiced across all facilities. However, a third of FDOs (31.25%) mentioned they were not linked to a referral mechanism in cases of medical emergencies. This was assessed on the basis that a facility-in-charge could describe facility plans for referral of patients and that an ambulance could be called for emergencies. Laboratories were present in 84.90% of visited health centres and 50% of FDOs (χ^2^ = 7.273, *p* = 0.007). Results reflect an MoH order (nr. 695, 2010 on Primary Health Care), which determines that laboratories should be present in all health centres, but not necessarily in FDOs. There was a pharmacy offering compensate medicines in 95.45% of localities with a health centre, and in 68.75% of FDO localities (X2 = 8.119, *p* = 0.004). Their absence was therefore mainly found in rural localities.

#### Availability of community outreach plans and services

Only a small majority of facilities had formal outreach visit plans that were available and used by the health facility (HC: 63.64%; FDO: 56.25%). However, most health facilities stated carrying out the following outreach activities on a monthly basis: regular home visits, occasional home visits (in case of emergency) and community sensitization (group information sessions).

### Health workforce

According to WHO, a well-performing health workforce is one which “works in ways that are responsive, fair and efficient to achieve the best health outcomes possible, given available resources and circumstances” [17].

#### Number of doctors, medical assistants, and staff responsible for community nursing

Across all visited facilities, there was at least one medical doctor, with the majority being present 5 or more days per week (HC: 93.18%; FDO: 81.25%). Medical assistants were also on staff in all facilities. This data however does not permit to say whether staffing levels were sufficient according to population needs. See Table 3 for an overview.

**Table 3:** Health workforce (±SD, *p < 0.05, **p < 0.01)

Health Workforce	Facility type		Region			
	HC	FDO	North	Centre	South	
Family doctors having received training on updated PHC guidelines, last 12 months, on 3 tracer diseases (%)						(N = 60) module a
Ischemic heart disease	77.27%	56.25%	50.00%	76.19%	85.71%	43
Diabetes	77.27%	62.50%	61.11%	71.43%	85.71%	44
Hypertension	81.82%*	56.25%*	61.11%	76.19%	85.71%	45
Knowledge score of family doctors on chronic disease management (mean score out of 13)	8.14 ± 1.84	8.06 ± 2.14	8.39 ± 1.98	7.38 ± 2.08	8.62 ± 1.47	60
						(*N* = 182) module c
Adherence to principles of clinical history taking and physical examination, (mean score out of 13)	11.04 ± 1.37	10.67 ± 1.64	10.53 ± 1.30**	10.90 ± 1.63	11.33 ± 1.30**	182
Adherence to clinical guidelines for Ischemic heart disease, (mean score out of 41)	26.78 ± 5.68	27.55 ± 6.74	28.00 ± 5.75	29.08 ± 5.55	24.08 ± 5.68	38
Adherence to clinical guidelines for Diabetes, (mean score out of 42)	27.39 ± 5.90	27.38 ± 8.46	29.36 ± 4.92	26.06 ± 8.50	27.05 ± 5.75	49
Adherence to clinical guidelines for Hypertension, (mean score out of 39)	26.13 ± 6.19	24.12 ± 6.04	26.18 ± 6.67	25.66 ± 6.32	25.03 ± 5.73	95

The perception of facility-in-charges on the importance of having medical assistants working at community level (community medical assistants) was mixed, with about a third mentioning the position was necessary and important, and a quarter mentioning the terms of reference should be reviewed by the MOH. About 15% of facility in charges viewed the position as unnecessary.

#### Training received on updated PHC guidelines (focus on 3 tracer diseases)

Family doctors at health centre level were more likely to have received training (in the last 12 months) on updated clinical guidelines for the study’s tracer diseases than at FDO level: ischemic heart disease (HC: 77.27%; FDO: 56.25%), diabetes (HC: 77.27%; FDO:62.50%) and hypertension (HC: 81.82%; FDO:56.25%, χ^2^ = 4.09, *p* = 0.043). Training on updated guidelines was mainly provided by neighbouring health centre personnel, followed by the State University of Medicine and Pharmacy.

#### Knowledge of chronic disease management

The knowledge of medical doctor/facility-in-charges on chronic disease management was assessed through five questions with a total possible score of 13.Results show an average knowledge level related to chronic disease management, with the mean score at 8.14 ± 1.84/13at health centre level, and 8.06 ± 2.14/13 at FDO level (Additional file 6: Knowledge of chronic disease management).

#### Adherence to principles of clinical history and physical examination

To assess the adherence to principles of clinical history taking, physical examination and adherence to clinical standards, family doctors were observed during consultations with clients for the 3 tracer diseases. Family doctors adhered well to the general principles of clinical history taking and patient care, with a general score of 10.94 out of 13. Results highlight some weaknesses in clinical consultations, with doctors scoring27.39/42 for diabetes consultations; 25.61/39 for hypertension; and 27.03/41 for ischemic heart disease. Only 26.8% doctors washed hands before procedures and 27.9% washed hands after the procedure. Female doctors tended to have a better score across these quality indicators. See Additional file 7: Adherence to principles of clinical history taking*.*

### Information

A well-functioning health information system (HIS) is one that “ensures the production, analysis, dissemination and use of reliable and timely information on health determinants, health systems performance and health status” [17]. In this study we only assessed the availability and quality of HIS.

#### Availability of a health information system, including patient records (paper and electronic)

A small majority of health centres used a ‘mixed system’ - both a paper and electronic system (63.64%), followed by a ‘paper only system’ for maintaining patient records (34.09%). Only 2.27% (*N* = 1) of health centres used solely an electronic system. FDOs tended to use a ‘paper system only’ for patient records (81.25%), followed by a ‘mixed system’ - both a paper and electronic system (18.75%). It is important to note that the electronic system in Moldova was only recently introduced. See Table 4 for an overview of findings.

**Table 4:** : Information(**p < 0.01)

Information	Facility type		Region			N
	HC	FDO	North	Centre	South	(=60)
Type of health information system at facility (%)						
Paper system only	34.09%**	81.25%**	55.56%	42.86%	42.86%	28
Electronic system only	2.27%	0.00%	0.00%	4.76%	0.00%	1
Both paper and electronic version	63.64%**	18.75%**	44.44%	52.38%	57.14%	31
Patient record system is well organised (paper) (%)	86.36%	87.50%	77.78%	85.71%	95.24%	52

#### Quality of patient record system and records (paper version)

The quality of the patient record system was assessed based on indicators related to the safety and confidentiality of storage units; a clear organisation system (by disease or alphabetical) and organised patient cards within the system. A majority of facilities had a well organised system (HC: 86.36%; FDO: 87.50%). When observing consultations (*N* = 179), results highlighted that health centre doctors tended to fill in the documents in a more comprehensive manner than doctors working in FDOs (86.15% versus 75.51%).

### Medical products, vaccines & technologies

This WHO building block refers to the "equitable access to essential medical products, vaccines and technologies of assured quality, safety, efficacy and cost-effectiveness, and their scientifically sound and cost-effective use [17].

#### Availability of essential medical equipment for NCDs

The availability of essential equipment was assessed based on the presence and functionality of 24 equipment items (see Table 5). Results show that there was an equipment gap in 24.24% of health centres and 36.72% of FDOs. A total of 20 institutions had non-functional equipment (7 in urban areas, 13 in rural areas); however, this equates to only 3.19% of the total equipment assessed (*N* = 46).

**Table 5:** Medical products, vaccines & technologies (*p < 0.05, **p < 0.01, ***p < 0.001)

Medical products, vaccines & technologies	Facility type		Region			N
	HC	FDO	North	Centre	South	Module a (=60)
Current list of compensated medicines for non-communicable diseases perceived as appropriate (%)	77.27%	75.00%	77.78%	85.71%	66.67%	46
Availability of essential medical equipment for NCDs, (cumulative score, %)	72.06%***	61.46%***	66.44%	70.24%	70.63%	N/A
Availability of essential medical supplies for NCDs, (cumulative score, %)	55.52%***	25.00%***	55.71%	43.21%	41.27%	N/A
	Pharmacy setting		Region			
	Urban	Rural	North	Centre	South	Module c (*N* = 54)
Availability of essential medicines, Cumulative score, (%)						
Diabetes medicine						
Available in all specific dosages	80.45%***	51.07%***	62.03%	67.05%**	55.88%**	N/A
Available at least in one dosage	97.22%***	68.95%***	76.47%	79.44%	82.35%	
Not available at all	2.78%***	31.05%***	23.53%	20.56%	17.65%	
Cardiovascular diseases medicine						
Available in all specific dosages	82.50%***	69.03%***	77.76%**	74.53%	69.67%**	N/A
Available at least in one dosage	93.44%***	83.82%***	90.44%*	83.75%*	88.60%	
Not available at all	6.56%***	16.18%***	9.56%*	16.25%*	11.40%	
Hypertension medicine						
Available in all specific dosages	86.48%***	74.81%***	82.39%***	82.26%***	72.17%***	N/A
Available at least in one dosage	97.08%*	92.16%*	94.12%	92.92%	95.10%	
Not available at all	2.92%*	7.84%*	5.88%	7.08%	4.90%	

#### Availability of essential medical supplies for NCDs

The availability of medical supplies was assessed based on the availability of 7 essential items. Results show a supply gap in 44.48% of health centres and 75% of FDOs.

#### Availability and adequacy of the list of essential medicine for NCDs

The list of essential medicines was available in nearly all facilities (HC: 100%; FDO: 93.75%). When asked about the adequacy of the current list of compensated medicines for non-communicable diseases, a quarter of facility-in-charges mentioned that the current list was only partially appropriate (HC: 22.73%; FDO: 25%). Stated reasons for the inadequacy of the list were mainly: the lack of full-compensation for medicines, the absence of other important chronic diseases from the list, and the lack of efficacy of some of the medicines.

#### Availability of essential NCD medicines

The official list of compensated medicines from the Ministry of Health was used for this assessment at the pharmacy level. For each tracer disease, a cumulative score was calculated based on the availability of medication and its various dosages. Results highlight a discrepancy between urban and rural localities, as well as a starker gap in the availability of diabetes medicines. It is important to note that pharmacies are private institutions that can decide on which drugs they will stock and sell.

#### Hypertension

The full set of compensated medicines for hypertension were found to be available in 86.48% of urban pharmacies, and in 74.81% of rural pharmacies. There was a total absence of essential hypertension medicine in 2.92% of rural pharmacies versus 7.84% of urban pharmacies.

#### Cardiovascular diseases

The full set of compensated medicines for diabetes were found to be available in 82.50% of pharmacies in urban localities, as compared to 69.03% of rural pharmacies. There was a total absence of essential cardiovascular medicine in 16.18% of rural pharmacies versus 6.56% of urban pharmacies.

#### Diabetes

The full set of compensated medicines for diabetes were found to be available in 80.45% of pharmacies in urban localities, as compared to only 51.07% of rural pharmacies. There was a total absence of essential diabetes medicine in 31.05% of rural pharmacies versus 2.78% of urban pharmacies.

### Financing

According to the WHO, a good health financing system “raises adequate funds for health, in ways that ensure people can use needed services, and are protected from financial catastrophe or impoverishment associated with having to pay for them” [17].

#### Adequacy of funding coordination and mechanisms for medicines compensation

A small majority of facility in-charges confirmed that the funding for NCDs from the National Health Insurance Company (NHIC) and the MoH is well coordinated at district and locality level (HC: 63.64%; FDO: 56.25%). According to respondent, the weaknesses were insufficient funding, and the inability of purchasing partially compensated medicines. However, 1 in 5 facility-in-charges thought that funding for NCDs was not well coordinated at district and locality level. About half of the respondents perceived the compensation mechanism for medicines to be partially adequate’ (HC: 54.55%; FDO: 43.75%). See Table 6 below.

**Table 6:** Financing

Financing	Facility type		Region			N (=60)
	HC	FDO	North	Centre	South	
Funding for NCDs from the NHIC and the MoH is well coordinated at the district level and localities	63.64%	56.25%	44.44%	71.43%	66.67%	37
Current mechanisms for medicines compensation are adequate	54.55%	43.75%	44.44%	57.14%	52.38%	31

### Leadership & governance

And finally, the leadership and governance building block involves “ensuring strategic policy frameworks exist and are combined with effective oversight, coalition-building, the provision of appropriate regulations and incentives, attention to system-design, and accountability” [17].

#### Participation and adequacy of quality improvement measures

Facility-in-charges were asked about the frequency of participation of their facility staff in peer review meetings for quality improvement at district level. A small majority of facilities participated in these meetings on a monthly basis (HC: 65.91%; FDO: 56.25%), followed by on a weekly basis (HC: 13.64%; FDO: 6.25%), and on a quarterly basis (HC: 11.36%; FDO: 12.50%). A notable 9% of health centres and 25% of FDOs mentioned never participating in these district-level meetings. See Table 7.

**Table 7:** Leadership and governance

Leadership & Governance	Facility type		Region			N (=60)
	HC	FDO	North	Centre	South	
Participation of health facilities in peer review meetings for quality improvement at district level (at least once a month, %)	79.55%	62.50%	66.67%	66.67%	90.48%	45
Availability of client feedback systems	81.8%***	37.5%***	77.8%	66.7%	66.7%	
Public display of the contact details of the MoH helpline for citizen complaints (%)	90.91%**	62.50%**	72.22%	76.19%	100.00%	50
Availability of information leaflets on the MoH helpline for citizens’ complaints (%)	75.00%	50.00%	72.22%	52.38%	80.95%	41
Availability of a box/book to collect public opinion on the quality of services (%)	81.82%***	37.50%***	77.78%	66.67%	66.67%	42

Participants were mainly specialised doctors and family doctors, followed by social workers, midwives and psychologists. Medical assistants and local government authorities were rarely present in these district level peer review meetings. The large majority of interviewed facility-in-charges from HCs and FDOs deemed these meetings to be important for improving the quality of care provided to patients.

#### Availability of client feedback systems

Mechanisms for client feedback on quality of services were present in a majority of health centres, but lacking at FDO level: Such mechanisms include: The public display of the contact details of the MoH helpline for citizen complaints (HC: 90.91%; FDO: 62.50%); information leaflets on the MoH helpline for citizens’ complaints (HC: 75%; FDO: 50%), and the availability of a box/book to collect public opinion on the quality of services (HC: 81.82%; FDO: 37.50%).

## Discussion

Results of this study highlight the broad range of strengths and weaknesses of the Moldovan primary health care system, as well as its potential to deliver NCD services that can ensure improved health (a), responsiveness (b), social and financial protection (c) and improved efficiency (d) (see Fig. 1 WHO Health Systems Framework).

Our findings highlight that service delivery (1), and information (3) were generally the strongest among the six WHO health systems building blocks. The availability of infrastructure, equipment, NCD services and a health information system were found in the majority of PHC facilities. These improvements provide a strong basis to further strengthen the quality of services, as well as their efficiency and accessibility.

In terms of the geographic distribution of NCD service provision, urban facilities generally fared better across all indicators.The urban-rural divide in NCD service provision is therefore still considerable in Moldova, although the situation seems to have improved since a 2007 survey [14].

*For improved health (a)***-** (see Fig. 1 WHO Health Systems Framework), sustained efforts are required to improve service quality in terms of staffing numbers, as well as their knowledge and skills, and investing further in health promotion activities. Health systems data from 2017 highlight that occupancy rates of family doctor and medical assistant positions are generally low and have decreased in the last 5 years [28]. Out migration is an issue, as well as an over-aged workforce. In 2014, the WHO noted that mid-level cadres (medical assistants) could be better harnessed to strengthen health promotion and play an active role in leading educational sessions both at facility and community level [8]. Although our data highlighted that training on clinical protocols occurred in a majority of HCs, knowledge on chronic disease management was found to be average across all facilities, as was the quality of clinical consultations. Further training, retention strategies, as well as promoting inter-disciplinary work seems essential to facilitate further progress and see improved health outcomes.

The second outcome of interest is *responsiveness (b)*, which WHO defines as ‘the ability of the health system to meet the population’s legitimate expectations regarding their interaction with the health system…’. [29] Our findings highlight that a culture of continuous quality improvement is not yet well established, albeit some quality improvement measures (quality circles at district level) were known, participation lacked regularity, as well as the presence of multidisciplinary staff. Mechanisms for social accountability (such as complaint boxes) were greatly lacking at FDO level, even though their presence is mandatory according to government policies [4]. Responsiveness of the health system could be further improved by strengthening accountability processes from national to local level, and by increasing inter-disciplinary work from community to district level, as well as further strengthening community outreach plans and referral systems.

And finally, an essential prerequisite to reach universal health coverage is *improved efficiency (d)*, defined as more value for money invested. Our study highlights that facility-in-charges have differing perceptions on the adequacy of funding and reimbursement mechanisms for NCDs – with about fifty-percent deeming the mechanisms as non-adequate. The lack of essential medical supplies and medicines at PHC level (especially in the case of diabetes) is also an area that requires continued attention – to ensure timely treatment of patients with chronic diseases, equity in access, and reducing catastrophic health expenditures from OOPs.

With regards to *social and financial protection(c)*, *this survey did not collect any data to report on these outcomes. Another parallel study that collected household data will be published at a later date* [30]*.*

### Limitations

The majority of data collected in this survey focused on service delivery and medical products (WHO building blocks 1 & 4). Our data on the other building blocks is therefore more limited. This survey did not collect any information on the use of data for decision-making or carry out an in-depth assessment of the use of the electronic health information system – although these are important components for the ‘Information building block’*.* Although the study aimed to carry out as much direct observation as possible in the time frame and budget available, some questionnaire items in survey modules (a) and (b) only rely on self-reporting of health professionals. This limitation will partly be lessened with the future publication of a household survey which was carried out in the same localities that took into account clients’ views on the availability of services and their quality.

## Conclusions

Our study provides a comprehensive update on the state of NCD service provision at primary health care level in Moldova, both in terms of coverage and quality of care. Although the main objective the study was to inform and evaluate an NCD programme, the findings are relevant to a broader community of policy makers, programme managers, and researchers working to improve service provision in the country. The study highlights that the basics of the family medicine model are in place in terms of infrastructure and equipment – while other aspects such as staffing and medicine availability remain rather critical and require urgent attention. The gaps in service provision identified require broad health system improvements to ensure NCD related policies and strategies are embedded in primary health care service provision. Ensuring universal health coverage, calls for integrated care and multisectoral collaboration at all levels of the public and private sectors, including education, social services, socio-cultural activities, local administration. For instance, community medical assistants could play an important role in the in the prevention and management of NCDs, working as liaison officers between the health system, the social welfare system and community members. In the light of the current PHC and public health reforms, strengthening the capacity of inter-sectoral collaboration at district health council level seems to be a valuable opportunity.

To maintain continuous quality improvement processes, strengthening pre- and in-service training could be further prioritised given the rise of NCDS and the shifting burden of disease –especially in rural areas and at primary care level. As per government policies and strategies, clinical protocols and evidence-based health promotion strategies should be further embedded within health facility processes with clear roles and support structures. Findings also highlight that the culture of continuous quality improvement of service delivery could be reinforced – including through closer collaboration with communities, meaningful quality circles, physician networks and peer review with a focus on evidence based remedial action through clear quality improvement and accountability mechanisms. The limited electronic information systems and administrative work remain a challenge and should be strengthened if integrated care is envisaged, and thus a broader multi-sectoral collaboration. Future research would be needed on the use of data for decision making within the health system – especially in terms of ensuring that life-saving equipment, medicine and staff are available.

## Additional files

Additional file 1: **Survey module a.** The file contains the blank English language copy of survey module (a). (PDF 451 kb)

Additional file 2: **Survey module b.** The file contains the blank English language copy of survey module (b). (PDF 280 kb)

Additional file 3: **Survey module c.** The file contains the blank English language copy of survey module (c). (PDF 207 kb)

Additional file 4: Infrastructure, cleanliness and maintenance. The file contains the full data collected on health facility infrastructure and maintenance. (PDF 171 kb)

Additional file 5: Water, sanitation and hygiene. The file contains the full data collected on the availability of water and sanitation infrastructure and hygiene measures at the health facility level. (PDF 174 kb)

Additional file 6: Knowledge of chronic disease management. This file contains the full results providers’ knowledge by area of chronic disease management. (PDF 60 kb)

Additional file 7: Adherence to principles of clinical history taking. This file contains all the data collected on clinical history taking during a consultation. (PDF 157 kb)

## Data Availability

The datasets used and/or analysed during the current study are available from the corresponding author on reasonable request.

## Abbreviations

CVD: Cardiovascular Disease
FDO: Family Doctor Office
HC: Health Centre
HIS: Health Information System
MHI: Mandatory Health Insurance
MOH: Ministry of Health
NCD: Non-Communicable Disease
NHIC: National Health Insurance Company
ODK: Open Data Kit
OOP: Out of Pocket Payment
PHC: Primary Health Care
SDC: Swiss Agency for Development and Cooperation
Swiss TPH: Swiss Tropical and Public Health Institute
UHC: Universal Health Coverage
WHO: World Health Organisation
