# Supplementary material for: Quality of care for non-communicable diseases in the Republic of Moldova: a survey across primary health care facilities and pharmacies
Source: BMC Health Serv Res. 2019 Jun 4;19:353. doi: 10.1186/s12913-019-4180-4 (PMC6547568; doi:10.1186/s12913-019-4180-4)
Supplement: Supplementary file 1 — Survey module a. The file contains the blank English language copy of survey module (a). (PDF 451 kb) [file 12913_2019_4180_MOESM1_ESM.pdf]

I) Quality of Care Assessment - Infrastructure Assessment
PHCs - Moldova

Survey Questionnaire
Version 13: 24.06.2017

Survey Information

Data collector and Location	Response
a. Facility Name	
b. Facility Type	**HC**<br>**OMF**
c. Raion name	Briceni<br>Cahul<br>Criuleni<br>Edineț<br>Fălești<br>Orhei<br>Ștefan Vodă<br>Taraclia<br>Ungheni<br>Vulcanesti<br>Cantemir<br>Căușeni<br>Cimișlia<br>Florești<br>Glodeni<br>Ialoveni<br>Leova<br>Rezina<br>Strășeni<br>Telenești
d. Raion ID -- *See list*	
e. Locality Name	

f. Locality ID - *See list*	
g. Interviewer ID	
start_time of interview	
Consent received	

PLEASE TAKE PICTURES OF THE FACILITY (OUTSIDE AND INSIDE), as well as PHARMACIES (OUTSIDE ONLY)

"Good morning/afternoon/evening. My name is _______________.
I am working as a data collector for the Healthy Life Project in Moldova. It is a project focusing on the prevention and control of non-communicable diseases in Moldova. It is implemented in coordination with the government, and led by the Swiss Tropical and Public Health Institute.
We are currently conducting a survey on the state of PHCs Moldova and would like to carry out an assessment of the infrastructure, equipment and processes that are available in this health centre".

The first part of the survey should be answered by the head of clinic (doctor).The head of the health facility can then assign you to another health staff to help you in completing this tool. The last section (medicines) should be completed at the nearest pharmacy. Please provide all your interlocutors with the information letter and ask for consent"

**SECTION A**		**SERVICE AVAILABILITY**		
	**Questions**	**Response**	**Skips**	**Source / Indicator**

1.	Which of the following **preventative PHC**services do you provide at this facility?<br><br>PLEASE SELECT ALL PREVENTATIVE SERVICES CURRENTLY ON OFFER IN THIS PHC	1. Promoting healthy lifestyles, inculcating skills in preventing and combating risk factors<br>2. Monitoring the physical and psychomotor development of the child<br>3. Vaccination<br>4. Prenatal care, observation of pregnant and post-natal care<br>5. Family planning<br>6. An annual medical examination with a prophylactic examination of persons over the age of 18 with the aim of preventing and early detection of diseases that are major in the morbidity and mortality structure<br>7. Periodic medical examinations of patients with diseases registered with a family doctor;<br>8. Provision of medical services to young people in accordance with applicable regulations;<br>9. Provision of services for early detection of pathology based on screenings<br>10. Others_______________________________		
2.	Which of the following **curative PHC** services do you provide at this facility?<br><br>*PLEASE SELECT ALL SERVICES CURRENTLY ON OFFER IN THIS PHC*	1. NCD consultation (primary examination, clinical examination, diagnosis, treatment)<br>2. Minor surgical interventions;<br>3. Medical treatment (intramuscular, intravenous procedures)<br>4. Medical and hygienic-dietary treatment, including compensated medicines and medical products<br>5. Monitoring of patients according to treatment plan<br>6. Referral to other services such as laboratory rapid tests, as well as physiotherapy and physical rehabilitation services,<br>7. Referral to a specialized physician in cases that are beyond the competence of the family doctor<br>8. Registration of patients with tuberculosis, confirmed by a specialized doctor<br>9. Medical care at home<br>10. Mental health services<br>11. Palliative care<br>12. Other (please specify)____________________		
3.	Do you carry out any outreach activities?			

	Note: Outreach activities include: any activity led by a nurse or doctor outside the walls of the PHC.	1. Yes<br>2. No<br>1. I do not know	*If No/I do not know, skip to question 5*	
4.	What types of outreach activities do you carry out on a regular basis in relation to <u>NCD patients</u> (at least once a month)*?*<br><br>*PLEASE READ OUT; AND SELECT ALL THAT APPLIES.*	1. Regular home visits (follow up on patients)<br>2. Home visits for persons that cannot move without assistance<br>3. Occasional home visits (in case of emergency)<br>4. Community sensitization (group information sessions)<br>5. Other (please specify………)		
5.	Do you have laboratory services in this PHC?	1. Yes<br>2. No		
6.	Is there at least one pharmacy in this locality offering compensated medicines?	1. Yes<br>2. No		
**SECTION B**				
7.	How many days of the working week is at least one doctor present to provide general primary health care services?	1. 1-2 days per week<br>2. 3-4 days per week<br>3. 5 or more days per week		
8.	How many doctors work at this facility?<br><br>THIS INCLUDES BOTH FAMILY DOCTORS AND SPECIALISTS.	------		
9.	How many nurses (medical assistants) work here ?	_____		
10.		1. Yes	***If no, skip to question12.***	

	Does this facility have a community medical assistant responsible for community nursing?	2. No		
11.	Is there a job description for the position of ' community medical assistant' at the facility?	1. Yes<br>2. No		
12.	What is your perception on this job description and/or position (of community health nurse)?<br><br>*PLEASE SUMMARISE THE ANSWER*	-------------------------------------		
13.	Does the facility have organisational charts and job descriptions supporting interdisciplinary family health teams?<br><br>HERE THE ANSWER IS LIKELY NO, AS THE PROJECT WOULD LIKE TO INTRODUCE THIS.	1. Yes<br>2. No		
14.	..<br>In your view what types of professionals should be included in interdisciplinary health teams?<br><br>PLEASE READ OUT THE RESPONSES, AND SELECT ALL THAT APPLY	1. Family doctor<br>2. Medical assistant<br>3. Specialised doctors<br>4. Midwives<br>5. Social workers<br>6. Physiotherapists<br>7. Psychologists/ Mental Health specialist<br>8. Homecare workers<br>9. Other (please specify)_____<br>10. I do not think there should be interdisciplinary health teams.<br>77. I don't know		
15.	Does the facility have an outreach visit plan related to NCDs (available and used) ?	1. Yes<br>2. No	***If no, skip to question 17***	
16.	Are the planned visits done in cooperation with other community services. i.e. social services?	1. Yes		

		2. No		
17.	Does the current workload on medical assistants enable them to also carry out community outreach and health promotion activities?	Always<br>Very Frequently<br>Occasionally<br>Rarely<br>Very Rarely<br>Never		
18.	Aside from administrative duties, do the medical assistants in this facility have enough time for clinical work (prevention, diagnosis, treatment)?	1. Always<br>2. Often<br>3. Occasionally<br>4. Rarely<br>5. Never		
19.	Does someone from this PHC participate in peer review meetings for quality improvement at the raion level?<br><br>*Note: quality improvement circles – an MoH policy to better link raion to PHC level. Please ask to see documents that demonstrate the participation in these meetings.*	1. Very Frequently (once a week)<br>2. Frequently (once a month)<br>3. Occasionally (every 3 months)<br>4. Rarely (twice a year or less)<br>5. Very Rarely (once a year or less)<br>6. Never<br>7. Quality circles/meetings do not exist	***If responses 6 or 7, skip question 20.***	
20.	What type of professionals usually attend these meetings?<br><br>SELECT ALL THAT APPLY	1. Specialised doctors<br>2. Family doctor<br>3. Midwives<br>4. Social workers<br>5. Nutritionists<br>6. Physiotherapists<br>7. Psychologists<br>8. Homecare workers<br>9. Other (please specify)		
21.	How would you rate the importance of these meetings in improving the quality of care you provide to your patients?	1. Very important<br>2. Important<br>3. Somewhat important<br>4. Not important<br>5. Not at all important<br>77. I don't know		
22.	Which staff member in this facility has the responsibility for health promotion at the community level?	1. Doctor<br>2. Medical assistants<br>3. other (please specify)_____	***If response 4, and 77 are selected skip to question 24***	

		4. There is no staff member with this responsibility<br>77. I don't know		
23.	What type of health promotion activities does the staff member carry out?<br><br>PLEASE LIST BRIEFLY KEY ACTIVITIES	________________________		
24.	In the last 12 months, how many staff (doctors and medical assistants) have been trained in Health Promotion ?	1. -----------<br>77. I don't know	***If answer is 0 or 77, skip to question 26.***	
25.	Who provided the training?<br><br>PLEASE STATE POSITION and INSTITUTION	1. ___________<br>77. I don't know		
26.	According to you, what is the definition of health promotion?<br><br>(PLEASE ASK FOR A LIST OF KEY COMPONENTS)	----------------------------		
27.	In the last 12 months, on which of the following updated PHC guidelines have the family doctors in this facility been trained on?<br><br>PLEASE READ OUT AND SELECT ALL THAT APPLY	1. Ischemic heart disease<br>2. Diabetes<br>3. Hypertension<br>4. No training received	***If answer 4, skip to question 30.***	
28.	Who provided the training?<br><br>PLEASE STATE POSITION and INSTITUTION	_____________<br>77. I don't know		
29.	How would you rate the quality of the training(s) ?	1. Very Good<br>2. Good<br>3. Acceptable<br>4. Poor<br>5. Very Poor		

30.	What treatments are possible for a case of atrial fibrillation that complicates a myocardial infarction?<br><br>(DO NOT READ THE RESPONSES, LET THE PERSON ANSWER. SELECT ALL ANSWERS MENTIONED)	1. Emergency cardioversion<br>2. Oxygen<br>3. Digoxin<br>4. Dopamine<br>5. Furosemide<br>6. The doctor does not mention any of the above options		
31.	During the physical examination of a hypertensive patient, for which signs/symptoms do you have to check for?<br>(DO NOT READ THE RESPONSES, LET THE PERSON ANSWER. SELECT ALL ANSWERS MENTIONED)	1. Signs suggestive of secondary hypertension<br>2. Signs suggestive of mental illness<br>3. Signs of dehydration<br>4. Evidence of visceral obesity<br>5. Signs of dyslipidemia<br>6. The doctor does not mention any of the above options		
32.	What are the risk factors for stable ischemic heart disease?<br><br>(DO NOT READ THE RESPONSES, LET THE PERSON ANSWER. SELECT ALL ANSWERS MENTIONED)	1. Level of systolic and diastolic blood pressure<br>2. Dyslipidemia<br>3. High junction glucose of 5.5 mmol / l<br>4. Old age<br>5. Male sex<br>6. Female sex<br>7. The doctor does not mention any of the above options		
33.	What are the hospitalization criteria for a type 2 diabetes patient?<br><br>(DO NOT READ THE RESPONSES, LET THE PERSON ANSWER. SELECT ALL ANSWERS MENTIONED)	1. Decompensation of DZ, requiring insulin therapy;<br>2. Precoma or diabetic coma (ketoacidosis, hypoglycaemia);<br>3. Progression of vascular complications;<br>4. The need to educate the patient with a disease state longer than 3 years<br>5. The need to educate the patient with the de novo diagnosis<br>6. The doctor does not mention any of the above options		
34.	What does it mean if glucose is at > 11.1 mmol / l 2 hours after a meal?	1. Healthy person<br>2. Diabetes gestational<br>3. Diabetes melitus<br>4. Decrease in glucose tolerance<br>5. Modification in basal blood glucose<br>6. The doctor does not mention any of the above options		

	(DO NOT READ THE RESPONSES, LET THE PERSON ANSWER. SELECT ALL ANSWERS MENTIONED)			
	**SECTION C**			
35.	The facility and immediate surroundings (facility yard, waiting area outside) are free from long grass, paper debris and solid waste	1. Yes<br>2. No		
36.	The facility has a rubbish bin which is properly used and not overflowing	1. Yes<br>2. No		
37.	There is a designated waiting room or area for patients.<br>***Note:*** *The waiting area does not have to be a separate room.*	1. Yes<br>2. No	***If no (2), skip to question skip to question 39***	
38.	The current waiting area is mopped, free of dust, trash; dirt, spider webs, and generally tidy.	1. Yes<br>2. No		
39.	There is at least one designated consulting room for women	1. Yes<br>2. No		
40.	There is at least one designated consulting room for children	1. Yes<br>2. No		
41.	All examination room(s) ensure(s) privacy/confidentiality (door, window blind, curtain).	1. Yes<br>2. No		

42.	All examination rooms are mopped, free of dust, trash; dirt, spider webs, and the rooms are generally tidy.	1. Yes<br>2. No		
43.	All examination rooms are well illuminated.	1. Yes<br>2. No		
44.	The facility has electricity<br><br>*Note: Electricity from the grid /supplier*	1. Yes<br>2. No	**If 2, skip to 46**	
45.	During the past 7 working days, did you have any power cuts of more than 1 hour during opening hours?	1. Yes<br>2. No		
46.	The facility has a functional generator	Yes<br>No		
47.	Does the facility have a functional communication equipment?<br>*Note: functional landline telephone*	Yes<br>No		
48.	The facility has functional computer.	Yes<br>No		
49.	The facility has a functional printer.	Yes<br>No		
50.	The facility has internet access.	Yes<br>No		

51.	During the past 7 working days did you have internet for at least 1 hour every day?	Yes<br>No		
52.	What type of patient record system does the facility use?	Paper system only<br>Electronic system only<br>Both paper and electronic version<br>No patient record system	**If 4,go to question 55**	
53.	Is the record system well organized?<br>*Note: can patient records be identified rapidly*	Yes<br>No		
54.	Are there sufficient empty patient record cards available?<br>*Note: at least 20 record cards*	Yes<br>No		
**SECTION D**				
55.	There is running water in the facility (out of the tap).	Yes<br>No	**If no skips to 58**	
56.	There is warm water available (out of the tap).	Yes<br>No		
57.	Functional washing points exist in examination rooms and/or entrance hall, and soap or hand disinfectants and water are available	Yes<br>No		
58.	Labeled containers for medical waste disposal are available in all required areas (e.g. examination rooms)	Yes<br>No		
59.	The facility has adequate and safe disposal of sharps (sharps box/container)	Yes		

		No		
60.	The facility has adequate and safe disposal of infectious waste.	Yes<br><br>No		
61.	Infectious waste is temporarily stored at a protected place<br><br>*Note: At the facility and inaccessible to non-authorized persons.*	Yes<br><br>No		
62.	Sharps waste is temporary stored at a protected place	Yes<br><br>No		
63.	There is regular and appropriate collection for infectious waste	Yes<br><br>No		
64.	There is regular and appropriate collection for sharps waste	Yes<br><br>No		
65.	The facility has essential disinfectants and antiseptics	Yes<br><br>No		
66.	The facility has chlorine solution or other disinfectants to disinfect contaminated instruments in all required areas (e.g. in examination rooms)	Yes<br><br>No		
67.	The facility has at least one accessible and functional toilet for patients	Yes<br><br>No		
68.	The facility has at least one accessible and functional toilet for staff.	Yes, separate toilet<br><br>Yes, but the same toilet as for patients<br><br>No		

69.	The toilet(s) or latrine is clean.<br><br>*Note: If there is more than one, all must be clean.*	Yes<br><br>No		
70.	A washing point is available near the toilet or latrine.	Yes<br><br>No		
71.	Soap is available at the washing point near toilet or latrine.	Yes, liquid soap<br><br>Yes, solid soap<br><br>No		
**SECTION E**				
72.	Is the facility location visibly displayed to the public?<br><br>*Note: There are signs in front of PHC indicating what the building is and what services are offered*	Yes<br><br>No		
73.	Are the facility opening hours visibly displayed to the public?<br><br>*Note: The opening hours are visibly displayed to the public (visibly outside the building)*	Yes<br><br>No		
74.	Is a contact phone number visibly displayed to the public?<br><br>*Note: A contact phone number is visibly displayed to the public (visibly outside the building)*	Yes<br><br>No		
75.	Are the tariffs visibly displayed to the public/patients?	Yes<br><br>No		

76.	Are the contact details of the Ministry of Health helpline for citizen complaints publicly displayed?	Yes<br>No		
77.	Are information leaflets about the Ministry of Health helpline for citizens' complaints available at the heath facility?	Yes<br>No		
78.	Is information on the prohibition of smoking displayed to the public?<br><br>*Note: Information openly displayed (e.g. waiting area, examination rooms, or outside)*	Yes<br>No		
79.	Is the Charter of Patient's Rights and Responsibilities visibly displayed in the waiting area?<br><br>*Note: Information openly displayed (e.g. waiting area, examination rooms, or outside)*	Yes<br>No		
80.	Does the facility have a box or mechanism to collect public opinion on the quality of services?<br><br>*Note: only select 'yes' if this mechanism is used.*	Yes<br>No		
81.	Does the facility have mechanisms to facilitate referral of emergency patients to the next level?<br><br>*Note: Health staff is able to describe facility plans for referral of patients – ambulance can be called. Using private cars is not a referral practice.*	Yes<br>No	If no, skip to the next section.	
82.	Is the referral system in line with government policy? (specific criteria)	Yes<br>No		
**SECTION F**				
83.		1. Yes, printed version only		

	Is the collection of standard protocols for PHC available at the facility?<br><br>*Note: the material needs to be easily accessed.*	2. Yes, electronic version only<br>3. Yes, both versions<br>4. No		
84.	Is the list of essential drugs for emergency situations available at the facility?<br><br>*Note: the material needs to be easily accessed.*	Yes<br><br>No		
85.	Is a health promotion plan and trained staff available at the PHC level?<br>(as required by the Public Health Department)<br><br>ASK TO SEE DOCUMENTS	1. Yes, both are in place<br>2. Yes, but only the plan<br>3. Yes, but only the staff<br>4. No	**If answer is 3 or 4, skip to question 88.**	
86.	Is this plan well understood and used?	1. Yes, fully<br>2. Yes, partly<br>3. No	If 1, skip to question 88.	
87.	Please explain your response:	----------------------------		
88.	Are the essential national clinical protocols on Hypertension available?<br><br>*Note: the material needs to be easily accessed.*	Yes<br><br>No		
89.	Are the essential national clinical protocols on Ischemic Heart Disease available?<br><br>*Note: the material needs to be easily accessed.*	Yes<br><br>No		
90.	Are the essential national clinical protocols on Diabetes available?<br>*Note: the material needs to be easily accessed.*	Yes<br><br>No		

**SECTION G**				
91.	Do you feel that the funding for NCDs from the NHIC and the MoH is well coordinated at the raion level and localities?	1. Yes, both at the raion and locality level<br>2. Yes, but only at raion level<br>3. Yes, but only at locality level<br>4. No<br>5. I do not know	**If 1 or 5, skip to question 93**	
92.	Please specify	______________		
93.	Do you consider the current mechanisms for medicines compensation to be adequate?	1. Yes, fully<br>2. Yes, partly<br>3. No	**If 1, skip to question 95**	
94.	Please specify	______________		
95.	Is the current list of compensated medicines appropriate for non-communicable diseases?	1. Yes, fully<br>2. Yes, partly<br>3. No	**If 1, skip to question 97**	
96.	Please specify	______________		
97.	Are disease prevention measures financially insured?	1. Yes, fully<br>2. Yes, partly<br>3. No	**If 1, skip to question 99**	
98.	Please specify	______________		
**SECTION H**				
99.	Posters or other materials onhealth promotion are visible to the public	1. Yes<br>2. No		
100.	Information on Vaccination/Immunization is visible to the public<br>*(current information)*	1. Yes<br>2. No		
101.		1. Alcohol abuse		

	Are there information leaflets for patients on the following topics:	2. Nutrition (prevention and awareness)<br>3. Hypertension<br>4. Cardiovascular diseases<br>5. Sport and activity<br>6. Smoking<br>7. Diabetes<br>8. There are no leaflets		
**SECTION I**				
**General medical equipment**				
102.	Minisurgery	Available, functional<br>Available, NOT functional<br>Not available		
103.	Nebulizer	Available, functional<br>Available, NOT functional<br>Not available		
104.	Ambu mask	Available, functional<br>Available, NOT functional<br>Not available		
105.	Strong source of light in good condition (portable)	Available, functional<br>Available, NOT functional<br>Not available		
106.	Nasal speculum	Available, functional<br>Available, NOT functional<br>Not available		
107.	Otoscope	Available, functional<br>Available, NOT functional<br>Not available		

108.	Ophtalmoscope	Available, functional<br>Available, NOT functional<br>Not available		
109.	Glucometer	Available, functional<br>Available, NOT functional<br>Not available		
110.	Peak flow meter<br><br>*Tube that checks vital lung functions and capacity*	Available, functional<br>Available, NOT functional<br>Not available		
111.	Tape measure	Available, functional<br>Available, NOT functional<br>Not available		
112.	Pen light	Available, functional<br>Available, NOT functional<br>Not available		
113.	Neurological hammer	Available, functional<br>Available, NOT functional<br>Not available		
114.	Weight scale for adults	Available, functional<br>Available, NOT functional<br>Not available		
115.	Sphygmomanometer for adults	Available, functional<br>Available, NOT functional<br>Not available		
116.	Stethoscope for adults	Available, functional<br>Available, NOT functional<br>Not available		
117.	Sterilization equipment and anti-septical protocol	Available, functional<br>Available, NOT functional<br>Not available		
118.	Pulse oximeter	Available, functional<br>Available, NOT functional<br>Not available		
119.	Refrigerator	Available, functional<br>Available, NOT functional		

		Not available		
120.	Thermometer	Available, functional<br>Available, NOT functional<br>Not available		
121.	Timer	Available, functional<br>Available, NOT functional<br>Not available		
122.	Snellen eye chart	Available, functional<br>Available, NOT functional<br>Not available		
123.	Test strips for Glucometer	Yes<br>No		
124.	Urine protein test strips	Yes<br>No		
125.	Urine ketones test strips	Yes<br>No		
126.	Urine microalbuminuria test strips	Yes<br>No		
127.	Fluorescein strips	Yes<br>No		
128.	Blood cholesterol assay or cholesterol express test	Yes<br>No		
129.	Lipid profile	Yes<br>No		
**Other equipment**				
130.	EKG machine	Available, functional<br>Available, NOT functional<br>Not available		
131.	Defibrillator	Available, functional<br>Available, NOT functional<br>Not available		
132.	Ultrasound machine	Available, functional<br>Available, NOT functional<br>Not available		
133.	GPS coordinates			

SECTION J				
	Is there a pharmacy in this locality?	Yes<br>Yes, but it is closed today<br>No	If, 2 or 3, skip to the end of the interview.	
	Name of pharmacy	_____________		
134.	Distance from facility<br><br>*(Facility that was assessed in this survey)*	Less than 100 m<br>Between 100-500m<br>Between 500m-2km<br>More than 2 km away		
135.	Does the person in charge of pharmacy accept to participate in this study (consent)?	Yes<br>No	If no (2), skip to the end of the interview.	
136.	ACIDUM ACETYLSALICYLICUM<br><br>*PLEASE SELECT ONLY DOSAGES AVAILABLE IN THE PHARMACY TODAY.*	75 mg tablets<br>100 mg tablets<br>325 mg tablets<br>None available		
137.	AMIODARONUM	200 mg<br>Not available		
138.	DIGOXINUM	0.25 mg tablets<br>Not available		
139.	ISOSORBIDI DINITRAS	Yes<br>No		
140.	ISOSORBIDI MONONITRAS<br><br>*PLEASE SELECT ONLY DOSAGES AVAILABLE IN THE PHARMACY TODAY.*	20 mg tablets<br>40 mg tablets<br>None available		
141.	WARFARINUM<br><br>*PLEASE SELECT ONLY DOSAGES AVAILABLE IN THE PHARMACY TODAY.*	3 mg tablets<br>5 mg tablets<br>None available		
142.	CLOPIDOGRELUM	75 mg tablets<br>None available		
143.	SIMVASTATINUM<br><br>*PLEASE SELECT ONLY DOSAGES AVAILABLE IN THE PHARMACY TODAY.*	10 mg tablets<br>20 mg tablets<br>40 mg tablets<br>None available		

144.	AMLODIPINUM<br><br>*PLEASE SELECT ONLY DOSAGES AVAILABLE IN THE PHARMACY TODAY.*	5 mg tablets<br>10 mg tablets<br>None available		
145.	BISOPROLOLUM<br><br>*PLEASE SELECT ONLY DOSAGES AVAILABLE IN THE PHARMACY TODAY.*	2.5 mg tablets<br>5 mg tablets<br>10 mg tablets<br>None available		
146.	ENALAPRILUM<br><br>*PLEASE SELECT ONLY DOSAGE AVAILABLE IN THE PHARMACY TODAY.*	5 mg tablets<br>10 mg tablets<br>20 mg tablets<br>None available		
147.	INDAPAMIDUM<br><br>*PLEASE SELECT ONLY DOSAGES AVAILABLE IN THE PHARMACY TODAY.*	1.5 mg tablets<br>2.5 mg tablets<br>None available		
148.	LISINOPRILUM<br><br>*PLEASE SELECT ONLY DOSAGES AVAILABLE IN THE PHARMACY TODAY.*	5 mg tablets<br>10 mg tablets<br>20 mg tablets<br>None available		
149.	LOSARTANUM<br><br>*PLEASE SELECT ONLY DOSAGES AVAILABLE IN THE PHARMACY TODAY.*	50 mg tablets<br>100 mg tablets<br>None available		
150.	RAMIPRILUM<br><br>*PLEASE SELECT ONLY DOSAGES AVAILABLE IN THE PHARMACY TODAY.*	5 mg tablets<br>10 mg tablets<br>None available		
151.	SPIRONOLACTONUM<br><br>*PLEASE SELECT ONLY DOSAGES AVAILABLE IN THE PHARMACY TODAY.*	25 mg tablets<br>50 mg tablets<br>None available		
152.	TORASEMIDUM<br><br>*PLEASE SELECT ONLY DOSAGES AVAILABLE IN THE PHARMACY TODAY.*	5 mg tablets<br>10 mg tablets<br>None available		
153.	VERAPAMILUM	80 mg tablets<br>Not available		

154.	METOPROLOLUM<br><br>*PLEASE SELECT ONLY DOSAGES AVAILABLE IN THE PHARMACY TODAY.*	25 mg tablets<br>50 mg tablets<br>100 mg tablets<br>None available		
155.	GLIBENCLAMIDUM<br><br>*PLEASE SELECT ONLY DOSAGES AVAILABLE IN THE PHARMACY TODAY.*	3.5 mg tablets<br>5 mg tablets<br>None available		
156.	GLICLAZIDUM<br><br>*PLEASE SELECT ONLY DOSAGES AVAILABLE IN THE PHARMACY TODAY.*	30 mg tablets<br>60 mg tablets<br>None available		
157.	METFORMINUM<br><br>*PLEASE SELECT ONLY DOSAGES AVAILABLE IN THE PHARMACY TODAY.*	500 mg tablets<br>850 mg tablets<br>1000 mg tablets<br>None available		
158.	REPAGLINIDUM<br><br>*PLEASE SELECT ONLY DOSAGES AVAILABLE IN THE PHARMACY TODAY.*	0.5 mg tablets<br>1 mg tablets<br>2 mg tablets<br>None available		
159.	INSULINUM HUMANUM (Cod ATC: A10AB01)<br><br>*PLEASE SELECT ONLY DOSAGES AVAILABLE IN THE PHARMACY TODAY.*	100 UI/ml – 3 ml - injectable solution<br>100 UI/ml – 10 ml - injectable solution<br>None available		
160.	INSULINUM HUMANUM (Cod ATC: A10AC01)<br><br>*PLEASE SELECT ONLY DOSAGES AVAILABLE IN THE PHARMACY TODAY.*	100 UI/ml – 3 ml – injectable suspension<br>100 UI/ml – 10 ml - injectable suspension<br>None available		
161.	INSULINUM HUMANUM (Cod ATC: A10AD01)<br><br>*PLEASE SELECT ONLY DOSAGES AVAILABLE IN THE PHARMACY TODAY.*	100 UI/ml – 3 ml - injectable suspension<br>100 UI/ml – 10 ml - injectable suspension<br>100 UI/ml – 3 ml –injectable pen<br>None available		

END				
162	**Thank you very much for the interview.**			
163	Interviewee (pharmacy) comments	----------------------		
164	Interviewer comments	------------------------		
165	GPS location of pharmacy			
166	End time of interview			
