# Supplementary material for: Quality of care for non-communicable diseases in the Republic of Moldova: a survey across primary health care facilities and pharmacies
Source: BMC Health Serv Res. 2019 Jun 4;19:353. doi: 10.1186/s12913-019-4180-4 (PMC6547568; doi:10.1186/s12913-019-4180-4)
Supplement: Supplementary file 2 — Survey module b. The file contains the blank English language copy of survey module (b). (PDF 280 kb) [file 12913_2019_4180_MOESM2_ESM.pdf]

II) Quality of Care Assessment – Clinical Observation
PHCs - Moldova

Survey Questionnaire
Version 12 : 19.06.2017

Survey Information

Data collector and Location	Response
Facility Name	
Facility Type	**HC**<br>**OMF**
Raion name	Briceni<br>Cahul<br>Criuleni<br>Edineț<br>Fălești<br>Orhei<br>Ștefan Vodă<br>Taraclia<br>Ungheni<br>Vulcanesti<br>Cantemir<br>Căușeni<br>Cimișlia<br>Florești<br>Glodeni<br>Ialoveni<br>Leova<br>Rezina<br>Strășeni<br>Telenești
Raion ID -- *See list*	
Locality Name	
Locality ID - *See list*	
Interviewer ID	
start_time of interview	
Consent received<br><br>*ONLY SELECT PATIENTS BETWEEN AGES 18-69 (born 1948 to 1999), and that have one of the following illnesses: diabetes, hypertension or ischemic heart disease.*	

Good morning! My name is [NAME]. We are here on behalf of the Healthy Life Project funded by the Swiss Development Cooperation to conduct a survey of selected health facilities of the project. The objective of this study is to assess the quality of health care services provided in health facilities. The Ministry of Health and ethical review board has approved this study.

Your experiences are very important to improve aspects related to quality of care. Information collected from this interview/questionnaire will be used by the Healthy Life Project. Summaries of findings might also be shared with municipal or national authorities.

The information collected from you will be kept confidential within the research team. Instead of using your name, we will give a non-personal identification number which cannot be traced individually. We are not interested in your disease or disease history but want to observe some aspects on the care that the doctor provides to you. You are free to decide whether or not you participate in this study. If you decide not to do it, there will be no negative effect.

→For this survey, we would need to consent of the doctor and the patient to observe the consultation. Please provide the person with the information letter and ask for consent.

SECTION 1		PROFILES OF HEALTH STAFF AND PATIENT			*Indicator 3.4*
	**Questions**	**Response**	**Skips**		
2	Doctors' gender	female<br>male			
5	Patients' gender	female<br>male			
6	Patients' year of birth<br><br>*(Note: only patients 18 to 69)*	______	*Ensure responses incude only 1948 to 1999.*		
SECTION 2		ADHERENCE TO CLINICAL HISTORY AND PROTOCOLS			
7	The medical doctor…<br><br>(PLEASE SELECT ALL THAT APPLY)	• … greets the client.<br>• … sees the client in privacy/confidentiality.<br>• … makes the client comfortable (e.g. seat offered)<br>• … asks the client about concerns, allows client to explain his/her health issue.<br>• … takes patient history (general history, specific to disease)<br>• … asks open ended questions during history taking<br>• … asks about any prescriptions the client is currently taking.<br>• … listens to the client and responds to client questions.<br>• …has the patient medical record at hand<br>• … uses the patient medical record for anamnesis<br>• ...fills in special prescription form<br>• …fills in medical record<br>• …uses information system to input data about patient			
8	For which illness(s) is the patient seen?	1. Hypertension<br>2. Ischemic heart disease<br>3. Diabetes	Only one selection possible.		

	Select the main illness/risk factor –on which the doctor will focus on				
9	For which type of visit is the patient seen?<br><br>SELECT ONLY ONE ANSWER	1. Diabetes-routine visit (stable glycemy)<br>2. Diabetes-non-routine visit (unstable glycemy)<br>3. Hypertension-routine visit (stable blood pressure)<br>4. Hypertension-non-routine visit (unstable blood pressure)<br>5. Ischemic heart disease routine visit (stable health situation)<br>6. Ischemic heart disease non-routine visit (unstable health situation)	Answers 1 or 2, skip questions 16-30 (= skip section 2.2. and 2.3).<br><br>Answers 3 or, 4, skip questions 10-17, and 23-30 (= skip section 2.1. and 2.3).<br><br>Answers 5 or, 6, skip questions 10-17 and 16-24, (= skip section 2.1. and 2.2).		
**2.1) Assessment of an adult diabetes mellitus patient**		Adherence to clinical assessment procedures, investigations and treatment guidelines			

10	The doctor asks questions on the illness about:<br><br>(PLEASE SELECT ALL THAT APPLY)	• … any specific health complaints<br>• ... general weakness<br>• ... urine discharge<br>• … vulvovaginitis or pruritus<br>• … appetite<br>• ... eye-sight<br>• … visit to opthalmologist<br>• ... alcohol<br>• ... smoking<br>• ... using other medicine<br>• … physical activity/sedentary way of life<br>• .... diet, including salt, sugar and fat intake			
11	The doctor asks questions about adherence with diabetes treatment	1. Yes<br>2. No<br>3. Not applicable / no medication needed			
12	The doctor conducts the following examinations…<br><br>(PLEASE SELECT ALL THAT APPLY)	• … checks blood pressure<br>• … weight measurement / calculation of body-mass index<br>• … of skin, mucus membranes, nodes of lymph, ears, nose, thyroid glands<br>• ... of eyes<br>• ... of chest, auscultation of lungs<br>• … auscultation of heart in 5 points<br>• ... of abdomen, palpation of liver and signs of percussion<br>• ... of legs (dry skin, the plantar callus, numbness, tingling, anesthesia, restless legs syndrome, foot deformities, fractures, arteritis, pain, ulcer and gangrene, injuries (wounds) difficult to treat, amputation)<br>• … perform physical examination according to patient complaints<br>• … and gives clear explanations to the client concerning the purpose of all examinations, tests and procedures.			

13	The doctor advises on and explains the following:<br>(PLEASE SELECT ALL THAT APPLY)	• … results of examinations<br>• ... the situation and diagnosis<br>• ... the prognosis<br>• … about needed examinations<br>• … nutrition, i.e. food intake and weight decrease<br>• … on the prevention and treatment of hypoglycemia and other acute and chronic complications of diabetes<br>• … on selfmonitoring - glycemia control and prevention of hypoglycaemia<br>• … about alcohol<br>• … about smoking<br>• … about physical exercise<br>• ... right ways of care of legs<br>• … potential complication of the illness<br>• … potential risks if illness is not treated<br>• … importance of adherence to treatment<br>• ... about follow-up visit			
14	The doctor advises on and explains the need for referral	1. Yes<br>2. No<br>3. Not applicable / not needed			
15	The doctor advises on and explains on prescribed medicines/treatment	1. Yes<br>2. No<br>3. Not applicable			
16	The doctor involves the patient in decision making	1. Yes<br>2. No<br>3. Not applicable			
17	The doctor answered the patient's questions	1. Yes<br>2. No<br>3. Not applicable / the patient did not have questions			

2.2 Assessment of an adult patient with hypertension		Adherence to clinical assessment procedures, investigations and treatment guidelines			
18.	The doctor asks questions on the illness about:<br><br>(PLEASE SELECT ALL THAT APPLY)	• … any specific health complaints<br>• … headache<br>• … the use of medicine other than for hypertension<br>• ... the use of contraceptives<br>• ... eye-sight<br>• … visit to opthalmalogist<br>• ... alcohol<br>• ... smoking<br>• … physical activity/sedentary way of life<br>• .... diet, including salt, sugar and fat intake			
19.	The doctor asks questions about high blood pressure	1. Yes<br>2. No<br>3. Not applicable			
20.	The doctor asks questions about adherence with hypertension treatment	1. Yes<br>2. No<br>3. Not applicable / not on treatment			
21.	The doctor conducts the following examinations…<br><br>(PLEASE SELECT ALL THAT APPLY)	• … checks blood pressure<br>• … weight measurement / calculation of body-mass index<br>• … of skin, mucus membranes, nodes of lymph, ears, nose, thyroid glands<br>• ... of eyes<br>• ... of chest, auscultation of lungs<br>• … auscultation of heart in 5 points<br>• ... of abdomen, palpation of liver and signs of percussion, palpation of kidneys<br>• ... of legs (change of color pale or rubor, cold skin, dry skin, edema, ulcers, paresthesia)<br>• … and gives clear explanations to the client concerning the purpose of all examinations, tests and procedures.			

22.	The doctor advises on and explains the following:<br>(PLEASE SELECT ALL THAT APPLY)	• … results of examinations<br>• ... the situation and diagnosis<br>• ... the prognosis<br>• … about needed examinations<br>• … about signs of extreme hypertension<br>• … about what to do when signs of extreme hypertension occur<br>• … nutrition, i.e. food intake<br>• … about alcohol<br>• … about smoking<br>• … about physical exercise<br>• … about oral contraceptives<br>• … potential complication of the illness<br>• … potential risks if illness is not treated<br>• …. adherence to treatment<br>• … follow-up visit			
23.	The doctor advises on and explains the need for referral	1. Yes<br>2. No<br>3. Not applicable			
24.	The doctor advises on and explains on prescribed medicines/treatment	1. Yes<br>2. No<br>3. Not applicable			
25.	The doctor involves the patient in decision making	1.Yes<br>2.No<br>3. Not applicable			
26.	The doctor answered the patient's questions	1. Yes<br>2. No<br>3. Not applicable			
**2.3 Assessment of a patient with ischemic heart disease**		Adherence to clinical assessment procedures, investigations and treatment guidelines			

27.	The doctor asks questions on the illness about:<br><br>(PLEASE SELECT ALL THAT APPLY)	• … any specific health complaints<br>• …heart pain<br>• …dyspnea<br>• … headache<br>• … the use of medicine other than for ischemic heart disease<br>• ... the use of contraceptives (female only)<br>• ... eye-sight<br>• … visit to ophthalmologist<br>• ... alcohol<br>• ... smoking<br>• … physical activity/sedentary way of life<br>• .... diet, including salt, sugar and fat intake				
28.	The doctor conducts the following examinations…<br>(PLEASE SELECT ALL THAT APPLY)	• … checks blood pressure<br>• … weight measurement / calculation of body-mass index<br>• … of skin, mucus membranes, nodes of lymph, ears, nose, thyroid glands<br>• ... of eyes<br>• ... of chest, auscultation of lungs<br>• … auscultation of heart in 5 points<br>• ... of abdomen, palpation of liver and signs of percussion, palpation of kidneys<br>• ... of legs (permanent sensation of cold legs, pale appearance, marbled teguments, edema, ulcers, paresthesia)<br>• … and gives clear explanations to the client concerning the purpose of all examinations, tests and procedures.….<br>• …performs medical examinations and other investigations as individually required (ex. ECG).				
29.	The doctor advises on and explains the following:<br>(PLEASE SELECT ALL THAT APPLY)	• … results of examinations<br>• ... the situation and diagnosis<br>• ... the prognosis<br>• … about needed examinations<br>• … about signs of heart attack<br>• … nutrition, i.e. liquid intake<br>• … about alcohol<br>• … about smoking<br>• … about physical exercise				

		• … about aspirin administration<br>• … potential complication of the illness<br>• … potential risks if illness is not treated<br>• …. adherence to treatment<br>• … follow-up visit			
30.	The doctor advises on and explains the need for referral	1. Yes<br>2. No<br>3. Not applicable			
31.	The doctor advises on and explains on prescribed medicines/treatment	1. Yes<br>2. No<br>3. Not applicable			
32.	The doctor advises on and explains on risk factors/health education	1. Yes<br>2. No<br>3. Not applicable			
33.	The doctor involves the patient in decision making	1. Yes<br>2. No<br>3. Not applicable			
34.	The doctor answered the patient's questions	1. Yes<br>2. No<br>3. Not applicable			
**2.4 Infection prevention and control measures**					
35	The doctor carried out the following prevention measures:<br><br>(PLEASE SELECT ALL THAT APPLY)	• … washed hands before the physical examination (including use of recommended desinfectant).<br>• … washed hands after the examination (including use of recommended desinfectant).			

**2.5 Record keeping and other**					
36	Does the doctor fully document the consultation in the patient card?	Yes, fully<br>Only partially<br>No			
37	Did the doctor have sufficient time for the patient?<br><br>*(i.e. does it seem that the doctor had enough time to answer questions, and that the patient had enough time to ask questions)*	Yes<br>No			
38	Was the consultation interrupted by someone else external to the consultation?	Yes<br>No			
39	Could anyone else see or hear the patient or doctor during the consultation?	Yes<br>No			
**INTERVIEW END**					
	**Thank you very much**				
	Interviewer comments<br><br>PLEASE ADD ANY COMMENT YOU THINK WOULD BE USEFUL FOR THE PROGRAMME TO KNOW.				

	End time of interview				
