# Supplementary material for: Quality of care for non-communicable diseases in the Republic of Moldova: a survey across primary health care facilities and pharmacies
Source: BMC Health Serv Res. 2019 Jun 4;19:353. doi: 10.1186/s12913-019-4180-4 (PMC6547568; doi:10.1186/s12913-019-4180-4)
Supplement: Supplementary file 3 — Survey module c. The file contains the blank English language copy of survey module (c). (PDF 207 kb) [file 12913_2019_4180_MOESM3_ESM.pdf]

III) QUALITY OF CARE ASSESSMENT – PATIENT RECORD STUDY PHCS - MOLDOVA

SURVEY QUESTIONNAIRE
VERSION 7:1 9.06.2017

Survey Information

Data collector and Location	Response
Facility Name	
Facility Type	**HC**<br>**OMF**
Raion name	Briceni<br>Cahul<br>Criuleni<br>Edineț<br>Fălești<br>Orhei<br>Ștefan Vodă<br>Taraclia<br>Ungheni<br>Vulcanesti<br>Cantemir<br>Căușeni<br>Cimișlia<br>Florești<br>Glodeni<br>Ialoveni<br>Leova<br>Rezina<br>Strășeni<br>Telenești
Raion ID -- *See list*	
Locality Name	
Locality ID - *See list*	
Interviewer ID	
start_time of interview	

SECTION 1		Patient records – with hypertension			
1.	**Questions**	**Response**		**Skips**	**Source**
2.	Sex	Male<br>Female	1<br>2		
3.	Age<br><br>*The patient must be between 18 and 69 – Born between 1948 and 1999.*	__________			
4.	Insurance coverage	Yes, and it is recorded<br>No, and it is recorded<br>No information in file<br>Unclear/unsure (poor recording)	1<br>2<br>3<br>4		
5.	Health condition (other than hypertension)	Diabetes<br>Ischemic heart disease<br>Other (please specify)________<br>No information in file<br>Unclear/unsure (poor recording)	1<br>2<br>3<br>4<br>5		
6.	Total number of visits for hypertension in the last 12 months	(please specify<br>No information in file<br>Unclear/unsure (poor recording)	1<br>2<br>3		
7.	Date on which diagnosis of hypertension was added to the patient health profile	(please specify)<br>No information in file<br>Unclear/unsure (poor recording)	1<br>2<br>3		
8.	Date on which treatment was first prescribed (for hypertension)	please specify)	1		

		No information in file<br>Unclear/unsure (poor recording)	2<br>3		
9.	Names of hypertension medication taken by the patient (if applicable) ?	1. ________________<br>No information in file<br>Unclear/unsure (poor recording)	1<br>2<br>3		
10.	Date on which last hypertension medication was prescribed	1. ________________<br>No information in file<br>Unclear/unsure (poor recording)	1<br>2<br>3		
11.	Which medication was prescribed (last prescription)	2. __________<br>No information in file<br>Unclear/unsure (poor recording)	1<br>2		
12.	Date on which last consultation occurred (linked to hypertension)	________________			
13.	Systolic blood pressure (linked to last visit)	________________<br>7777 if "No information in file" and 8888 if "Unclear/unsure (poor recording)"			
14.	Diastolic blood pressure (linked to last visit)	________________<br>7777 if "No information in file" and 8888 if "Unclear/unsure (poor recording)"			
15.	Weight (linked to last visit)	--------<br>7777 if "No information in file" and 8888 if "Unclear/unsure (poor recording)"			
16.	BMI (linked to last visit)	--------			

		7777 if "No information in file" and 8888 if "Unclear/unsure (poor recording)"			
17.	Waist circumference (linked to last visit)	---------<br>7777 if "No information in file" and 8888 if "Unclear/unsure (poor recording)"			
18.	Is the patient recorded as pregnant during (linked to last visit)	Yes<br>No<br>Unclear/don't know	1<br>2<br>3	This question should only appear if answer was 2, under question 2 (sex).	
19.	Height	______ (cm)<br>7777 if "No information in file" and 8888 if "Unclear/unsure (poor recording)"			
20.	In the past 12 months, has the cho-lesterol been measured by a doctor or other health worker?	Yes, and results are noted<br>Yes, but results are not in file<br>No information in file<br>Unclear/don't know	1<br>2<br>3<br>4		
21.	Is the patient a smoker?	Yes, and it is recorded<br>No, and it is recorded<br>No information in file<br>Unclear/unsure (poor recording)	1<br>2<br>3<br>4	If, 2, 3, 4, skip to question 23	
22.	In the last 12 months, has the doctor offered advice on quitting smoking?	Yes, and it is recorded<br>No information in file<br>Unclear/unsure (poor recording)	1<br>2<br>3		

23.	Has the patient been diagnosed as having an issue with alcohol abuse?	Yes, and it is recorded<br>No, and it is recorded<br>No information in file<br>Unclear/unsure (poor recording)	1<br>2<br>3<br>4		
24.	In the last 12 months, has the doctor offered advice on alcohol consumption?	Yes, and it is recorded<br>No information in file<br>Unclear/unsure (poor recording)	1<br>2<br>3		
25.	In the last 12 months, has the doctor offered advice on nutrition and diet?	Yes, and it is recorded<br>No information in file<br>Unclear/unsure (poor recording)	1<br>2<br>3		
26.	In the last 12 months, has the patient participated in a 'school of patients' session on hypertension at the facility?	Yes, and it is fully recorded (both signatures)<br>Yes, and it is partially recorded (only signature of doctor)<br>No (form is empty)<br>Unclear/unsure (poor recording)	1<br>2<br>3<br>4		
27.	In the last 12 months, has the doctor offered advice on physical activity?	Yes, and it is recorded<br>No information in file<br>Unclear/unsure (poor recording)	1<br>2<br>3		
28.	Was this medical record stored in an organized manner?	Yes<br>No	1<br>2		
29.	Is the quality of the record keeping satisfactory in this medical record?	Very satisfactory<br>Satisfactory<br>Unsatisfactory<br>Very unsatisfactory	1<br>2<br>3<br>4		

30.	Is there a mention of the family history of hypertension in the file?	Yes, and it is well recorded<br>Yes, but incomplete<br>No information in file	1<br>2<br>3		
31.	Interviewer comments:<br><br>PLEASE ADD ANY COMMENT YOU THINK WOULD BE USEFUL FOR THE PROGRAMME TO KNOW.	x			
