# Supplementary material for: Quality of care for non-communicable diseases in the Republic of Moldova: a survey across primary health care facilities and pharmacies
Source: BMC Health Serv Res. 2019 Jun 4;19:353. doi: 10.1186/s12913-019-4180-4 (PMC6547568; doi:10.1186/s12913-019-4180-4)
Supplement: Supplementary file 4 — Infrastructure, cleanliness and maintenance. The file contains the full data collected on health facility infrastructure and maintenance. (PDF 171 kb) [file 12913_2019_4180_MOESM4_ESM.pdf]

Additional file 4. Infrastructure, cleanliness and maintenance

	Facility Type		Region			
	HC	FMO	North	Centre	South	N
**The facility and immediate surroundings (facility yard, waiting area outside) are free from long grass, paper debris and solid waste.**						
Yes	90.91%	100.00%	88.89%	100.00%	90.48%	56
No	9.09%	0.00%	11.11%	0.00%	9.52%	4
**The facility has a rubbish bin which is properly used and not overflowing.**						
Yes	95.45%	100.00%	94.44%	100.00%	95.24%	58
No	4.55%	0.00%	5.56%	0.00%	4.76%	2
**There is a designated waiting room for patients.**						
Yes	97.73%	100.00%	100.00%	100.00%	95.24%	59
No	2.27%	0.00%	0.00%	0.00%	4.76%	1
**The current waiting area is mopped, free of dust, trash; dirt, spider webs, and generally tidy.**						
Yes	95.35%	100.00%	100.00%	95.24%	95.00%	57
No	4.65%	0.00%	0.00%	4.76%	5.00%	2
**There is at least one designated consulting room for women.**						
Yes	100.00%	93.75%	94.44%	100.00%	100.00%	59
No	0.00%	6.25%	5.56%	0.00%	0.00%	1
**There is at least one designated consulting room for children.**						
Yes	88.64%	81.25%	83.33%	90.48%	85.71%	52
No	11.36%	18.75%	16.67%	9.52%	14.29%	8
**All examination room(s) ensure(s) privacy/confidentiality (door, window blind, curtain).**						
Yes	95.45%	81.25%	83.33%	90.48%	100.00%	55
No	4.55%	18.75%	16.67%	9.52%	0.00%	5

**All examination rooms are mopped, free of dust, trash; dirt, spider webs, and the rooms are generally tidy.**						
Yes	95.45%	100.00%	94.44%	95.24%	100.00%	58
No	4.55%	0.00%	5.56%	4.76%	0.00%	2
**All examination rooms are well illuminated.**						
Yes	90.91%	87.50%	83.33%	85.71%	100.00%	54
No	9.09%	12.50%	16.67%	14.29%	0.00%	6
**The facility has electricity**						
Yes	100.00%	100.00%	100.00%	100.00%	100.00%	60
No	0.00%	0.00%	0.00%	0.00%	0.00%	0
**During the past 7 working days, did you have any power cuts of more than 1 hour during opening hours.**						
Yes	52.27%*	18.75%*	44.44%	33.33%	52.38%	26
No	47.73%	81.25%	55.56%	66.67%	47.62%	34
**The facility has a functional generator**						
Yes	29.55%	6.25%	16.67%	28.57%	23.81%	14
No	70.45%	93.75%	83.33%	71.43%	76.19%	46
**Does the facility have a functional communication equipment ?**						
Yes	100.00%	100.00%	100.00%	100.00%	100.00%	60
No	0.00%	0.00%	0.00%	0.00%	0.00%	0
**The facility has functional computer.**						
Yes	100.00%	93.75%	94.44%	100.00%	100.00%	59
No	0.00%	6.25%	5.56%	0.00%	0.00%	1
**The facility has a functional printer.**						
Yes	97.73%***	68.75%***	83.33%	95.24%	90.48%	54
No	2.27%	31.25%	16.67%	4.76%	9.52%	6
**The facility has internet access.**						
Yes	100.00%	75.00%	88.89%	100.00%	90.48%	56
No	0.00%	25.00%	11.11%	0.00%	9.52%	4
**During the past 7 working days did you have internet for at least 1 hour every day?**						

Yes	100.00%	100.00%	100.00%	100.00%	100.00%	56
No	0.00%	0.00%	0.00%	0.00%	0.00%	0

*p<0.05, *** p<0.001
