# Supplementary material for: Quality of care for non-communicable diseases in the Republic of Moldova: a survey across primary health care facilities and pharmacies
Source: BMC Health Serv Res. 2019 Jun 4;19:353. doi: 10.1186/s12913-019-4180-4 (PMC6547568; doi:10.1186/s12913-019-4180-4)
Supplement: Supplementary file 5 — Water, sanitation and hygiene. The file contains the full data collected on the availability of water and sanitation infrastructure and hygiene measures at the health facility level. (PDF 174 kb) [file 12913_2019_4180_MOESM5_ESM.pdf]

Additional file 5. Water, sanitation and hygiene

	Facility Type		Region			N
	HC	FMO	North	Centre	South	N =60
**Availability of running water in the facility (out of a tap).**						
Yes	100.00%	87.50%	94.44%	95.24%	100.00%	58
No	0.00%	12.50%	5.56%	4.76%	0.00%	2
**Availability of warm water (out of the tap).**						
Yes	63.64%	64.29%	52.94%	80.00%	57.14%	37
No	36.36%	35.71%	47.06%	20.00%	42.86%	21
**Availability of functional washing points in examination rooms and/or entrance hall, and soap or hand disinfectants and water**						
Yes	88.64%	78.57%	82.35%	85.00%	90.48%	50
No	11.36%	21.43%	17.65%	15.00%	9.52%	8
**Labelled containers for medical waste disposal are available in all required areas (e.g. examination rooms).**						
Yes	97.73%	87.50%	94.44%	90.48%	100.00%	57
No	2.27%	12.50%	5.56%	9.52%	0.00%	3
**The facility has adequate and safe disposal of sharps (sharps box/container).**						
Yes	97.73%	93.75%	100.00%	90.48%	100.00%	58
No	2.27%	6.25%	0.00%	9.52%	0.00%	2
**The facility has adequate and safe**						

**disposal of infectious waste.**						
Yes	97.73%	87.50%	94.44%	90.48%	100.00%	57
No	2.27%	12.50%	5.56%	9.52%	0.00%	3
**Infectious waste is temporary stored at a protected place.**						
Yes	90.91%	87.50%	94.44%	90.48%	85.71%	54
No	9.09%	12.50%	5.56%	9.52%	14.29%	6
**Sharps waste is temporary stored at a protected place.**						
Yes	90.91%	87.50%	94.44%	90.48%	85.71%	54
No	9.09%	12.50%	5.56%	9.52%	14.29%	6
**There is regular and appropriate collection for infectious waste.**						
Yes	93.18%	93.75%	94.44%	85.71%	100.00%	56
No	6.82%	6.25%	5.56%	14.29%	0.00%	4
**There is regular and appropriate collection for sharps waste.**						
Yes	93.18%	93.75%	94.44%	85.71%	100.00%	56
No	6.82%	6.25%	5.56%	14.29%	0.00%	4
**The facility has essential disinfectants and antiseptics.**						
Yes	100.00%	87.50%	94.44%	95.24%	100.00%	58
No	0.00%	12.50%	5.56%	4.76%	0.00%	2
**The facility has chlorine solution or other disinfectants to disinfect contaminated instruments in all required areas (e.g. in**						

**examination rooms).**						
Yes	88.64%	68.75%	77.78%	76.19%	95.24%	50
No	11.36%	31.25%	22.22%	23.81%	4.76%	10
**The facility has at least one accessible and functional toilet for patients.**						
Yes	93.18%	87.50%	88.89%	90.48%	95.24%	55
No	6.82%	12.50%	11.11%	9.52%	4.76%	5
**The facility has at least one accessible and functional toilet for staff.**						
Yes, separate toilet	65.91%*	37.50%*	44.44%	66.67%	61.90%	35
Yes, but the same toilet as for patients	27.27%	43.75%	44.44%	23.81%	28.57%	19
No	6.82%	18.75%	11.11%	9.52%	9.52%	6
**The toilet(s) or latrine is clean.**						
Yes	77.27%	62.50%	66.67%	66.67%	85.71%	44
No	22.73%	37.50%	33.33%	33.33%	14.29%	16
**A washing point is available near the toilet or latrine.**						
Yes	81.82%	68.75%	72.22%	80.95%	80.95%	47
No	18.18%	31.25%	27.78%	19.05%	19.05%	13
**Soap is available at the washing point near toilet or latrine.**						
Yes, liquid soap	63.64%	50.00%	55.56%	66.67%	57.14%	36
Yes, solid soap	2.27%	12.50%	5.56%	0.00%	9.52%	3

No	34.09%	37.50%	38.89%	33.33%	33.33%	21

*p<0.05
