# Supplementary material for: Quality of care for non-communicable diseases in the Republic of Moldova: a survey across primary health care facilities and pharmacies
Source: BMC Health Serv Res. 2019 Jun 4;19:353. doi: 10.1186/s12913-019-4180-4 (PMC6547568; doi:10.1186/s12913-019-4180-4)
Supplement: Supplementary file 6 — Knowledge of chronic disease management. This file contains the full results providers’ knowledge by area of chronic disease management. (PDF 60 kb) [file 12913_2019_4180_MOESM6_ESM.pdf]

Additional file 6. Knowledge of chronic disease management

Knowledge area	Correct response items	Facility Type		Region			
		HC	FMO	North	Center	South	Total
		Column N %	Column N %	Column N %	Column N %	Column N %	Count
Knowledge of atrial fibrillation	Emergency cardioversion	29.55%	6.25%	27.78%	23.81%	19.05%	14
	Digoxin	63.64%	81.25%	55.56%	71.43%	76.19%	41
Knowledge examination of a hypertensive patient	Signs suggestive of secondary hypertension	90.91%	75.00%	94.44%	71.43%*	95.24%*	52
	Evidence of visceral obesity	65.91%	62.50%	88.89%*	47.62%*	61.90%	39
Knowledge stable ischemic heart disease	Level of systolic and diastolic blood pressure	86.36%	81.25%	83.33%	76.19%	95.24%	51
	Dyslipidemia	79.55%	81.25%	72.22%	76.19%	90.48%	48
	High junction glucose of 5.5 mmol / l	34.09%	56.25%	44.44%	57.14%*	19.05%*	24
	Old age	47.73%	43.75%	61.11%	28.57%	52.38%	28
Knowledge hospitalization criteria type 2 diabetes	Decompensation of DZ, requiring insulin therapy;	79.55%	87.50%	94.44%	66.67%	85.71%	49
	Precoma or diabetic coma (ketoacidosis, hypoglycaemia);	88.64%	100.00%	94.44%	85.71%	95.24%	55
	Progression of vascular complications;	79.55%	75.00%	61.11%*	76.19%	95.24%*	47
	The need to educate the patient with the de novo diagnosis	4.55%	0.00%	5.56%	0.00%	4.76%	2
Knowledge mean glucose	Diabetes melitus	63.64%	56.25%	55.56%	57.14%	71.43%	37

*p<0.05
