# Supplementary material for: Quality of care for non-communicable diseases in the Republic of Moldova: a survey across primary health care facilities and pharmacies
Source: BMC Health Serv Res. 2019 Jun 4;19:353. doi: 10.1186/s12913-019-4180-4 (PMC6547568; doi:10.1186/s12913-019-4180-4)
Supplement: Supplementary file 7 — Adherence to principles of clinical history taking. This file contains all the data collected on clinical history taking during a consultation. (PDF 157 kb) [file 12913_2019_4180_MOESM7_ESM.pdf]

Additional file 7. Adherence to principles of clinical history taking, physical examination

	**Gender of medical doctor**		**Facility Type**		**Region**			**N (module b)**
	Male	Female	HC	FMO	North	Centre	South	N =180
**Adherence to principles of clinical history taking and physicalexamination (Score out of 13)**	10.60	11.04	11.04	10.67	10.53	10.90	11.33	180
The medical doctor:								
… greets the client.	97.62%	99.29%	99.25%	97.96%	100.00%	98.41%	98.44%	180
… seesthe client in privacy/confidentiality	83.33%*	94.29%*	93.23%	87.76%	87.27%*	88.89%	98.44%*	167
… makes the client comfortable (e.g. seat offered)	88.10%	94.29%	95.49%*	85.71%*	92.73%	93.65%	92.19%	169
… asks the client about concerns, allows client to explain his/her health issue.	95.24%	99.29%	99.25%	95.92%	98.18%	98.41%	98.44%	179
… takes patient history (general history, specific to disease)	59.52%	64.29%	62.41%	65.31%	52.73%*	60.32%	75.00%*	115
… asks open ended questions during history taking	57.14%	70.00%	66.92%	67.35%	52.73%***	61.90%*	84.38%***	122
… asks about any prescriptions the client is currently taking.	97.62%	96.43%	97.74%	93.88%	98.18%	93.65%	98.44%	176
… listens to the client and responds to client questions.	97.62%	96.43%	96.24%	97.96%	98.18%	96.83%	95.31%	176
… has the patient medical record at hand	97.62%	99.29%	99.25%	97.96%	100.00%	98.41%	98.44%	180
… uses the patient card for anamnesis	85.71%	90.71%	88.72%	91.84%	83.64%	88.89%	95.31%	163
… fills in special prescription form	80.95%	81.43%	84.21%	73.47%	83.64%	82.54%	78.13%	148
… fills in medical record	100.00%	99.29%	99.25%	100.00%	100.00%	98.41%	100.00%	181
… uses information system to input data about patient	19.05%	19.29%	21.80%	12.24%	5.45%**	30.16%**	20.31%	35

p<0.05,p<0.01, ***p<0.001*
